# Antibody response to oral biofilm is a biomarker for acute coronary syndrome in periodontal disease

**DOI:** 10.1038/s42003-022-03122-4

**Published:** 2022-03-04

**Authors:** Mariliis Jaago, Nadežda Pupina, Annika Rähni, Arno Pihlak, Helle Sadam, Nihal Engin Vrana, Juha Sinisalo, Pirkko Pussinen, Kaia Palm

**Affiliations:** 1 Protobios Llc, Mäealuse 4, 12618 Tallinn, Estonia; 2 Department of Chemistry and Biotechnology, Tallinn University of Technology, Akadeemia tee 15, 12618 Tallinn, Estonia; 3 Spartha Medical, 14B Rue de la Canardiere, 67100 Strasbourg, France; 4 Heart and Lung Center, Helsinki University Hospital, and Helsinki University, Helsinki, Finland; 5 Oral and Maxillofacial Diseases, University of Helsinki, FI-00014 Helsinki, Finland

**Keywords:** Biomarkers, Applied immunology

## Abstract

Cumulative evidence over the last decades have supported the role of gum infections as a risk for future major cardiovascular events. The precise mechanism connecting coronary artery disease (CAD) with periodontal findings has remained elusive. Here, we employ next generation phage display mimotope-variation analysis (MVA) to identify the features of dysfunctional immune system that associate CAD with periodontitis. We identify a fine molecular description of the antigenic epitope repertoires of CAD and its most severe form - acute coronary syndrome (ACS) by profiling the antibody reactivity in a patient cohort with invasive heart examination and complete clinical oral assessment. Specifically, we identify a strong immune response to an EBV VP26 epitope mimicking multiple antigens of oral biofilm as a biomarker for the no-CAD group. With a 2-step biomarker test, we stratify subjects with periodontitis from healthy controls (balanced accuracy 84%), and then assess the risk for ACS with sensitivity 71–89% and specificity 67–100%, depending on the oral health status. Our findings highlight the importance of resolving the immune mechanisms related to severe heart conditions such as ACS in the background of oral health. Prospective validation of these findings will support incorporation of these non-invasive biomarkers into clinical practice.

## Abstract (web summary)

This study finds that antibodies against antigens present in oral biofilm are potential biomarkers of acute coronary syndrome (ACS), the most severe form of coronary artery disease. By testing antibody response to periodontal pathogens, the authors find that patients with ACS have little or no immune response to a particular antigen epitope.

## Introduction

Coronary artery disease (CAD) is the leading cause of morbidity and mortality worldwide^1^ caused by metabolic disorders in lipid oxidation promoting inflammatory alterations on the endothelium^2,3^ and culminating in plaque rupture^4,5^. The heritability of CAD and its familial clustering are well established^6^. Genome-wide association studies (GWAS) have identified a number of causal CAD-associated genes and loci^7^. These findings highlight the largely polygenic nature of the inheritability of CAD^8,9^, rendering some individuals more susceptible or resilient to developing atherosclerosis^10^. CAD has been associated with the unhealthy lifestyle placing it among “immunoinflammatory” diseases^11^. While remarkable progress has been made in understanding the mechanisms of atherogenesis as robust methods of identifying high‐risk atherosclerosis via genomics and imaging are at hand, highly sensitive and specific biomarkers for CAD have remained elusive. Importantly, as much as 30% of control populations are thought to unknowingly include subjects with CAD, impacting power and accuracy of clinical biomarker studies^12–14^.

Periodontitis, a major oral dysbiosis-driven inflammatory disease, is associated with increased risk of atherosclerotic cardiovascular diseases^15^. Up to 700 bacterial species have been identified in the oral cavity (Human Microbiome Project Consortium). Intriguingly, DNA of periodontal pathogens (e.g., *Porphyromonas gingivalis*) and live bacteria have been detected in atherosclerotic lesions^16–18^. The microbial composition of gut microflora of patients with CAD has been found to be more inflammatory than in healthy patients^19^. Similarly, the oral microbiome of CAD patients may be altered^20^. Host-microbe interaction in the periodontium can initiate or even aggravate atherosclerotic processes through the activation of innate immunity, bacteremia, and direct involvement of cytokines and inflammatory proteins of oral microbiota^21–23^.

Recent research suggests that abnormal changes to the gut microbiota flora may also contribute extensively to the progression of CAD^24^. As the microbiome plays a central role in the balance between immune activation and immune tolerance^25^ and in the light of dysbiosis in microflora, it is no surprise that atherosclerosis has a strong autoimmune component^26^. First, CAD risk locus includes the major histocompatibility complex (MHC) containing a dense cluster of genes involved in inflammation, immunity, and self‐recognition^21,27^. Furthermore, a depletion of T or B cells leads to an attenuation of atherosclerosis^22^. Although T cells seem to be essential, B cells and antibodies play an accelerating and perpetuating role^23^. Thus, atherosclerosis is a chronic inflammatory disease with an autoimmune component^26^. Antibodies against oxidized low-density lipoproteins (oxLDL) positively correlated with the disease^28^. Besides oxLDL/ApoB, heat shock proteins (HSPs) and some foreign peptides from pathogens including cytomegalovirus (CMV), hepatitis C virus (HCV), HIV, human papillomavirus (HPV), and others have been proposed as atherosclerosis-relevant antigens^29^. However, the relation between antibodies and atherosclerotic disease burden and progression has remained unclear.

Here we used MVA^30,31^, an unbiased, high‐throughput, comprehensive approach based on next-generation phage display, to identify biomarkers of periodontal conditions associated with stable coronary artery disease and progression to acute coronary syndrome.

## Results

### Shared immunoreactivity to epitopes linked to periodontal pathogens

**Fig. 1:**
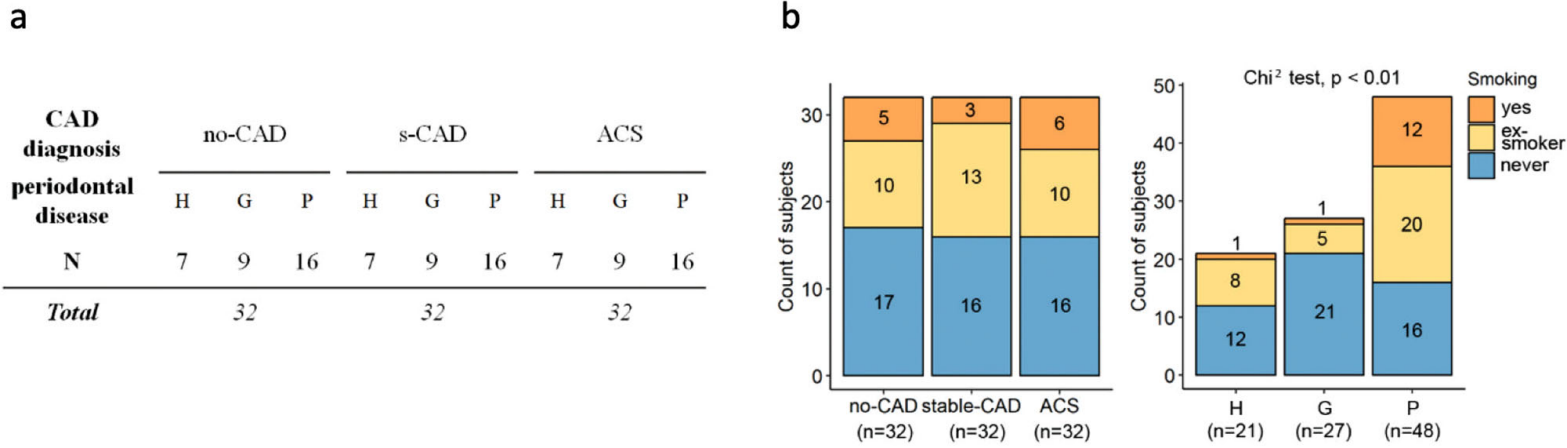
Characteristics of the clinical cohort. **a** Subjects divided in three groups based on CAD diagnosis (no-CAD, stable-CAD (s-CAD), or ACS). *Total*, size of the group. Each of the three CAD groups included subjects with different periodontal health diagnosis: periodontally healthy (H), patients with gingivitis (G) – a transient gum inflammation –, or patients with periodontitis (P). *N*, size of the sub-group. **b** Significant association between periodontal diagnosis and smoking is observed in the study cohort. Frequency distribution graphs of subjects (*n* = 96) in groups with coronary artery health condition (no-CAD, stable-CAD, or ACS) or periodontal condition (H, G, P). Statistically significant difference in prevalence of cigarette smoking was observed in periodontal condition groups, being highest among the periodontitis group subjects, but not within CAD groups (Chi^2^ test, *p* value < 0.05, *n* = 96 independent subjects). *x-axes – clinical subgroups; y-axes – number of subjects; color-fill – yes*, active cigarette smoker *(orange); ex-smoker*, has quit cigarette smoking *(yellow); never*, no history of cigarette smoking *(blue)*.

We used MVA immunoprofiling analysis of sera samples of 96 individuals from the Corogene cohort^32^ to identify peptide antigens related to antibody immune response in periodontal disease and CAD. Characteristics of the subjects according to their CAD and periodontal status are presented in Fig. 1 (Fig. 1a, Supplementary Table S1). Within the cohort, the proportion of ex- or active smokers was significantly higher in patients with periodontitis than in the rest of the cohort (Chi^2^, *p* < 0.01, Supplementary Table S1, Fig. 1b, Supplementary Fig. S2).

**Fig. 2:**
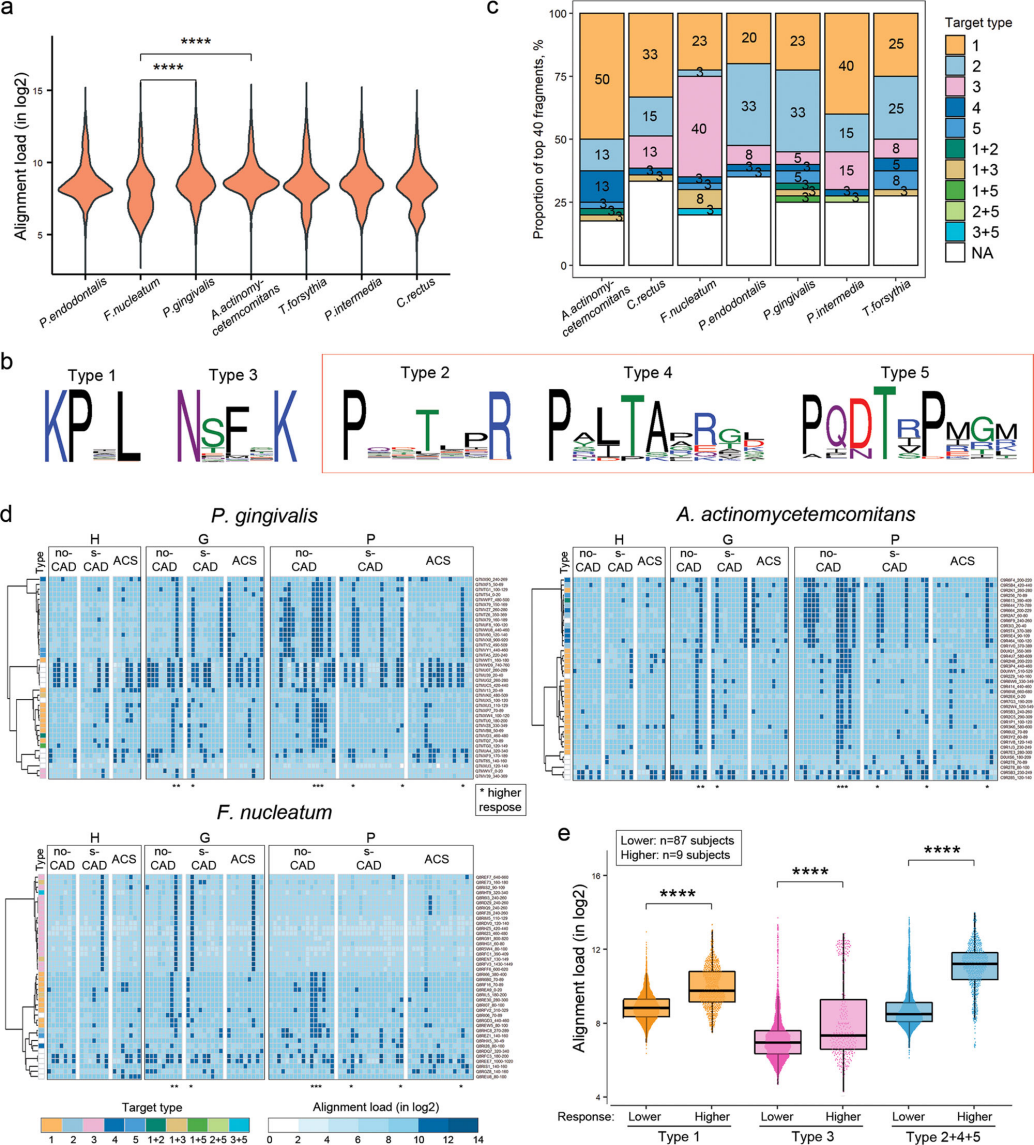
Shared immunoreactivity to epitopes linked to periodontal pathogens detected by MVA from immunoprofiles of the study cohort. Group-wide top 8088 epitopes from the MVA immunoprofiles were aligned onto proteomes of the most common periodontal pathogens (*n* = 7). Based on how many epitopes aligned and how enriched were the epitopes in MVA immunoprofiles for individual sample separately, top 40 fragments with highest alignment loads were selected per pathogen. **a** Antibody response to top 40 antigens for *P. gingivalis* and *A. actinomycetemcomitans* was the highest, whereas antibody response to *F. nucleatum* antigens was found to be low. Pair-wise Mann–Whitney *U*, two-sided, ****p < 0.0001, *p* values not adjusted for multiple comparisons. *y-axis* – alignment load, representing how many MVA immunoprofile epitopes aligned onto protein sequences and how abundantly were they seen in MVA immunoprofiles (in log2). **b** Unsupervised clustering identified most abundant epitopes with consensus sequences from alignments on target fragments. *Type 1*: KP.L in 1033 fragments; *type 2*: P..T.[P]R in 833 fragments; *type 3*: N[ST]F.K in 421 fragments; *type 4*: P[AYS][LI]TA.[REQ][GT][LDK] in 150 fragments; *type 5*: PQ[DN]T[RIV]P[MIR][GRT][MRK] in 107 fragments. *Outline* – type 2, 4, and 5 epitopes share similar core pattern P..T.[P][R]. **c** Proportions of antigenic epitope types across the antigens of the seven periodontal pathogens. *y-axis* – proportion (%) among top 40 antigen fragments (cumulative), *data labels on bars* - proportion (%), *x-axis* – oral pathogen species, *target type (fill color)* – epitope pattern type (from sequences in **b**). **d** Shared antibody response patterns to bacterial antigen epitopes (*target types, clustering on left*) were observed as specific for certain clinical heart and dental conditions (*grouping on top*). Immunoreactivity profiles against top 40 antigens in *F. nucleatum*, *P. gingivalis*, and *A. actinomycetemcomitans* are shown. Profiles were clustered row-wise according to epitope types (*type*, sequences in **b**). Subjects (in *lanes*) with highest immunoreactivity to top antigens (Supplementary Fig. S5) are marked with asterisks (*) under intensity plots. *Vertical lanes* – individual samples (*n* = 96), categorized based on CAD (no-CAD, s-CAD, ACS) and periodontal diagnoses (periodontally healthy (H), gingivitis (G), periodontitis (P)); *rows* – each row represents a distinct 20-aa antigenic region of protein primary sequence; *blue color-scale* – intensity of blue represents the alignment load of individual sample; *target type* – epitope type (sequences in **b**). **e** Distribution of subjects with low (*n* = 87) or high (*n* = 9) immune response (by Supplementary Fig. S5) to different targeted epitopes. Taken together, Types 2, 4, and 5 were more common to high-response subjects, as compared to other target types. Pair-wise Mann–Whitney *U*, two-sided, ****p < 0.0001, *p* values not adjusted for multiple comparisons. *y-axis* – alignment load in log2, *response* – high-response subjects (*n* = 9) or low-response subjects (*n* = 87), *type* – target epitope *type* (sequences in **b**).

Altogether we identified 14.5 million distinct peptide epitopes from the MVA immunoprofiles across the whole study cohort and converged these to 8088 most abundant and shared antigenic epitopes by clustering analysis (Supplementary Fig. S3). Given the gum disease background of the samples^33,34^, we first examined whether we could detect from the immunoprofiles of the study samples the immunoreactivity to the 7 most common periodontal pathogens (*Porphyromonas gingivalis*, *Tannerella forsythia*, *Prevotella intermedia*, *Fusobacterium nucleatum*, *Campylobacter rectus*, *Aggregatibacter actinomycetemcomitans*, and *Porphyromonas endodontalis*). Data analysis showed that antibody response to antigens of *P. gingivalis* and *A. actinomycetemcomitans* was the highest, whereas *F. nucleatum* antigens were on average significantly less recognized (Mann–Whitney *U*, ****p < 0.0001, two-sided) (Fig. 2a). By analyzing the sequences of the bacterial antigens, several dominant core epitopes shared by these antigens were identified, among which multiple types were derivatives of a common signature P.T.[P][R] (*Types 2, 4 and 5*), Fig. 2b, Supplementary Table S2). Interestingly, P..T.[P][R] patterns (i.e., Type 2, 4, or 5) were present in 45% and 30% of *P. gingivalis* and *A. actinomycetemcomitans* immunodominant antigens, respectively, whereas only 10% among *F. nucleatum* antigens (Fig. 2c). Shared antibody response patterns to bacterial antigen epitopes (*target types*, *clustering on left*) were observed as specific for certain clinical heart and dental conditions (*grouping on top*) (Fig. 2d, Supplementary Fig. S4). Notably, the majority of subjects (5/9) with high immune reactivity to these pathogenic periodontal bacteria (*indicated with dots under intensity plot*) belonged to the no-CAD group (Fig. 2d, Supplementary Fig. S5). Overall, in subjects (*n* = 9) with the highest immunoreactivity to these pathogens, P..T.[P][R] containing-epitopes (Types 2, 4, and 5) were the most prevalent and on average recognized at higher levels as compared to epitopes of Type 1 and 3 (Fig. 2e). Altogether, these data showed that immune response against a dominant core epitope P..T.[P][R], which was associated with common pathogenic oral bacteria, correlated with a differential risk of ACS.

### Different clinical groups share common features in the immunoprofiles

**Fig. 3:**
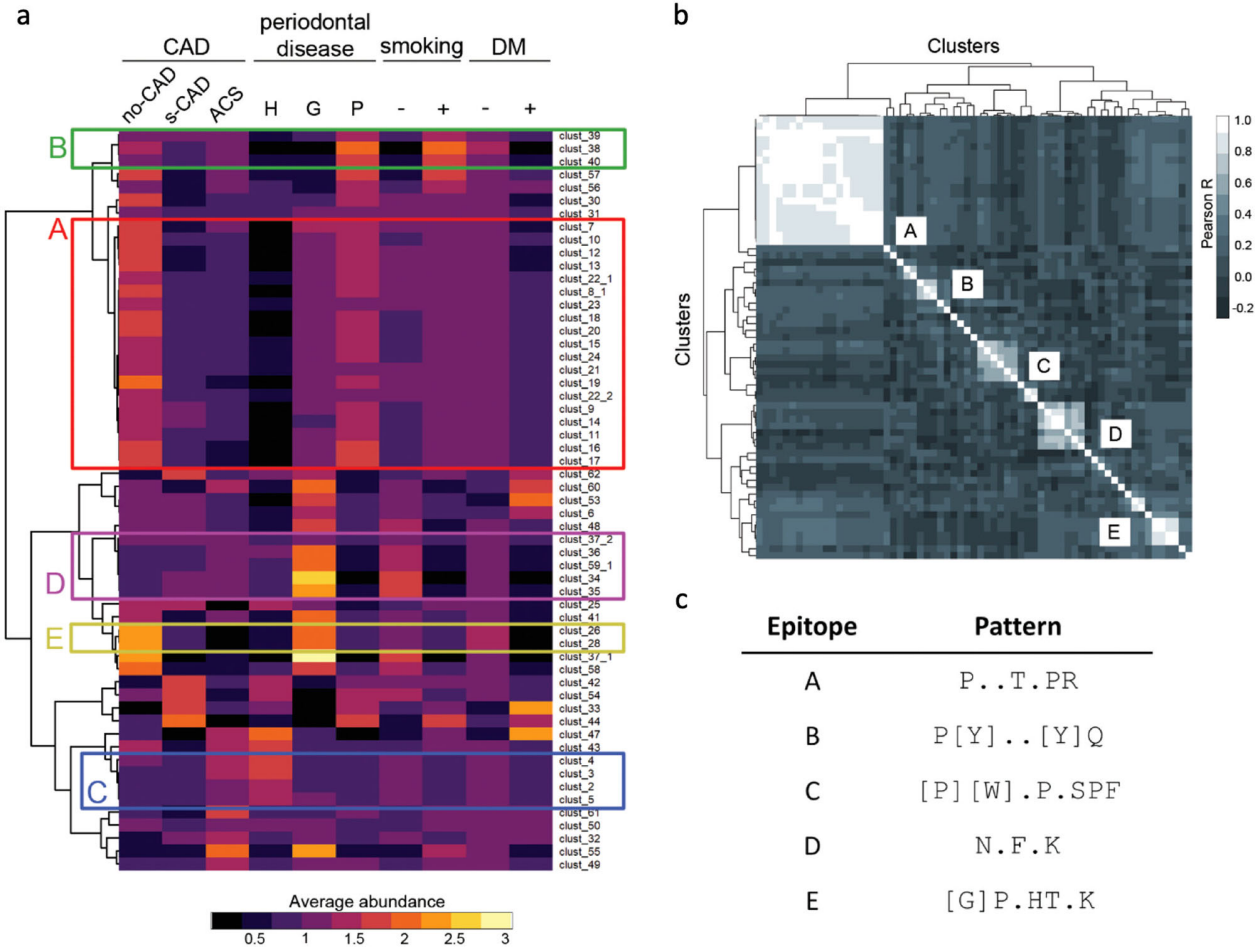
MVA immunoprofiles of subjects with different CAD conditions, periodontal disease severity, cigarette smoking behavior, and diabetes diagnosis. **a** 62 epitope clusters with group-specific MVA immunoprofile features across clinical classifiers. Although 19% of the subjects were diagnosed with diabetes (either type I or type II, not specified further), no significant association of diabetes with either CAD or periodontal diagnosis was found (Supplementary Fig. S2). *Average abundance*, calculated as mean of peptides containing the epitope within a given group and normalized with the mean values across all groups (color-coded from purple to yellow). Colored outlines with capital letters refer to epitopes in panel **c**. Clustering distance: Pearson correlation coefficient; clustering method: ward.D2. 57/62 clusters with high mean abundance (>150) across groups are shown. “−” under smoking designates subjects without any exposure to smoking, “+” depicts subjects with a history of exposure (ex-smoker) or currently actively smoking. CAD -coronary artery disease, no-CAD - no CAD diagnosis, s-CAD - stable CAD diagnosis, ACS - acute coronary syndrome, H -periodontally healthy controls, G - patients with gingivitis diagnosis, P - patients with periodontitis diagnosis, DM - diabetes mellitus. **b** Similar behavior-based clustering of 62 epitope clusters (*x- and y-axis*) using peptide abundance values (in log10) across the study cohort. Pearson R correlation indices (*color-scale*) were calculated and visualized in a correlation matrix. Five distinct large clusters were identified and defined as epitopes A to E. **c** Core consensus patterns of epitopes A to E, identified in Supplementary Fig. S7.

Given the findings that the immune response against oral pathogenic bacteria was associated with the clinical diagnosis, we next analyzed the difference of immune response to each of the 8088 epitopes in CAD or periodontal disease groups using ROC analysis with specific criteria: sensitivity ≥ 50% and specificity ≥ 70%, Kruskal–Wallis test *p* < 0.05. Clustered analysis based on sequence similarity arrived at 62 group-differentially targeted epitope clusters with shared core patterns (Supplementary Fig. S6, Supplementary Table S3). When we correlated the 62 epitope clusters based on average abundances in clinical diagnosis groups, clusters with similar response patterns were seen grouping together (Fig. 3a). Pearson correlation-based analysis united the 62 clusters further into five major epitopes (A to E), where the largest epitope A shared the core pattern P..T.PR (Fig. 3b, c, Supplementary Fig. S7). The epitope pattern P..T.PR includes P..T.[P][R], the one also observed as predominant among periodontal pathogens (Fig. 2b, Supplementary Table S2). We found that differential antibody response patterns against epitopes A to E were diagnosis group-specific (Fig. 3a, b, Supplementary Figs. S7 and S8). Specifically, stronger response to epitope A was specific to periodontitis and no-CAD cohorts (*red outline*), whereas stronger response to epitope B was detected in periodontitis and smoking subgroups (*green outline*) (Fig. 3a, b, Supplementary Fig. S8a, b). Stronger antibody response to epitope C, on the other hand, was characteristic to periodontally healthy patients but with an ACS diagnosis (*blue outline*), epitope D was more targeted in subjects with gingivitis (*yellow outline*), and epitope E in subjects with gingivitis but not in CAD (*pink outline*) (Fig. 3a, b, Supplementary Fig. S8c–e). In conclusion, these five major epitopes (A-E) were targeted by the strongest and differential antibody response across diagnosis groups.

### Microbial mimicry of the P..T.PR core epitope that is common to periodontitis encompasses the highly antigenic epitope of EBV VP26

**Fig. 4:**
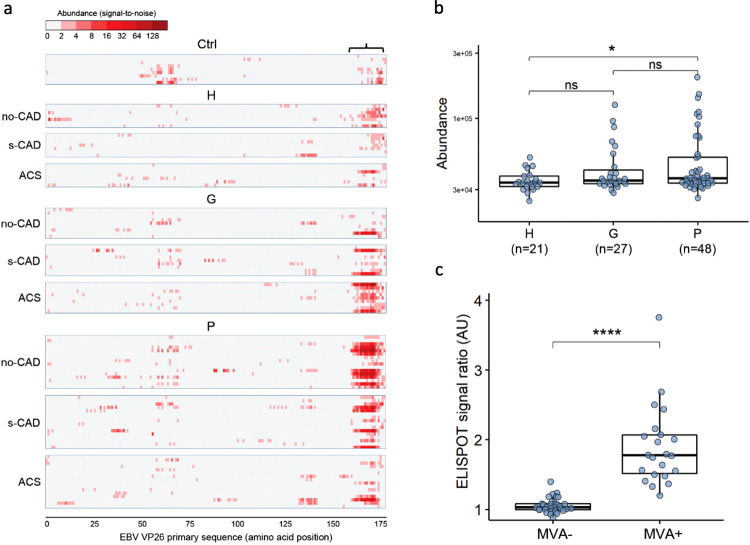
Immunoreactivity to epitope on VP26 EBV and alike mimicking features stratifies patients with periodontitis. **a** Individual immunoreactivity profiles (*n* = 96) of the study cohort against EBV protein VP26, shown for different clinical groups: periodontally healthy (H), gingivitis (G), or periodontitis (P). All peptides from individual immunoprofiles were aligned (with ≥ 6 matching amino acid positions) to primary sequence of EBV VP26 protein along with random reference. Peptide epitope abundance (as signal-to-noise ratio, *red color-scale*) is visualized per subject (*in rows*, separated into periodontal diagnosis groups) and per amino acid position (on *x-axis*). *Ctrl* – samples with negative EBV-viral capsid antigen serology (*n* = 9). **b** Highly antigenic P..T.PR epitope is differentially targeted across periodontal groups shown as box plots of abundance of peptides containing the epitope (Mann–Whitney *U*, two-sided, *p* values not corrected for multiple comparisons, *p < 0.05, ns *p* > 0.05, *n* = 96 independent patients), Supplementary File S6. **a**, **b** Abbreviations and group sizes: H periodontally healthy (*n* = 21 patients (3 × 7)), G gingivitis (n = 27 (3 × 9)), P periodontitis (*n* = 48 (3 × 16)), no-CAD no CAD diagnosis (*n* = 32), s-CAD stable CAD (*n* = 32), ACS acute coronary syndrome (*n* = 32). **c** Anti-P.DT.PR epitope-like immune response identified by MVA was in high correlation with data from independent dot ELISA validations. Phage particles displaying peptides with P.DT.PR or mutant P.DA.PR sequences were used for dot ELISA. Of the 52 randomly tested samples, those with a positive signal to anti-P.DT.PR response detected by MVA (>1200 abundance of P.DT.PR containing peptides, *MVA*+) showed significantly higher signals in dot ELISA (*y-axis, signal-to-background arbitrary unit (AU)*) compared to samples with a negative response from MVA data (*MVA-*, Mann–Whitney *U* test, two-sided, ****p < 0.0001, *n* = 52 independent subjects).

Our analyses highlighted a strong response to epitope A with the core pattern P..T.PR (Figs. 2d, 3c), which we have previously mapped to EBV VP26 protein encompassing 153-176aa^30,31^. When aligning all individual MVA immunoprofile-derived peptides of the current study cohort to the primary sequence of EBV VP26, we observed that subjects within the periodontitis group exhibited high immunoreactivity to this C-terminal epitope (Mann–Whitney *U*, *p < 0.05, two-sided, Fig. 4a, b). Independent validation was performed using dot ELISA analysis, where sera samples from the current clinical cohort were exposed to phage particles displaying the C-terminal VP26 epitope sequence (Fig. 4c). Patients predicted as seropositive against the P..T.PR epitope by MVA (MVA+) were observed with significantly higher immunoreactivity to the displayed EBV VP26 epitope in the dot ELISA analysis (Fig. 4c, Mann–Whitney *U*, ****p < 0.0001, two-sided). Therefore, these results further confirmed the MVA findings of specific seroreactivity mapping of epitope A (with the core pattern P..T.PR) to EBV VP26 (Fig. 4c). As we found that many antigens of the periodontal bacteria shared features of the epitope A (Fig. 2b, d, Supplementary Table S2) and could thus mimic the epitope of EBV VP26 antigen, we determined using annotation analysis that these could include a transmembrane protein signal peptidase I (100-129 aa, Uniprot accesssion Q7MTG1) and a cytosolic transcription termination factor Rho (160-189 aa, Q7MX79) of *P. gingivalis*, and isoleucine-tRNA ligase (770-789aa, C9R644) in *A. actinomycetemcomitans* (Supplementary Table S2). Also, epitope B was annotated to the tandem repeat (3 × 13 aa) in the C-terminal part (741-779 aa) of EBV nuclear antigen 6 (EBNA6) protein (Supplementary Fig. S9), in harmony with other studies reporting this region as highly immunogenic^35,36^. In conclusion, strong antibody response to EBV, in particular to VP26 and sequence-mimicking bacterial antigens, was distinguishing subjects with periodontitis from periodontally healthy controls.

### Biomarkers to predict ACS risk from periodontal disease

**Fig. 5:**
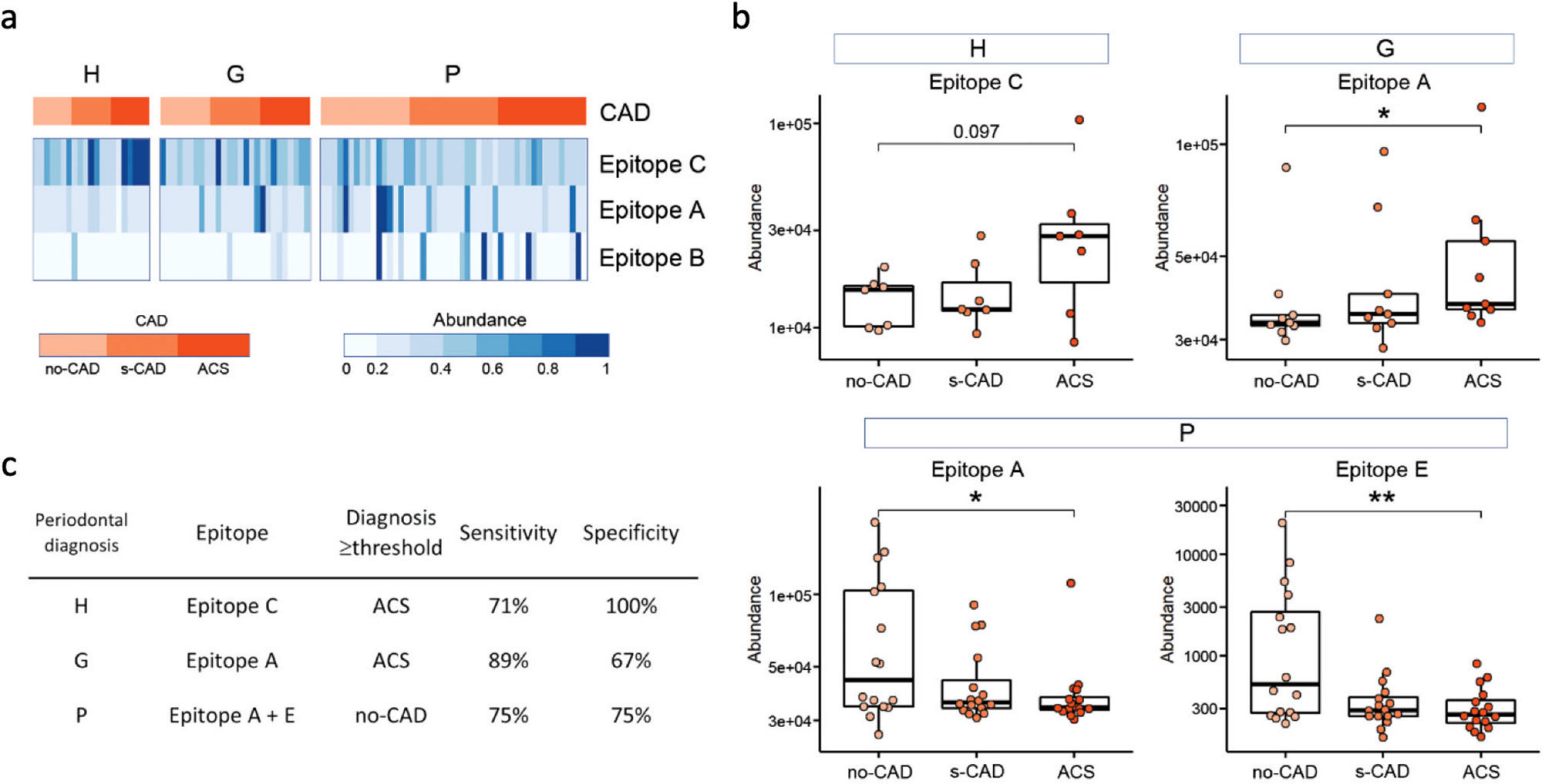
Immune response to epitope biomarkers to predict the ACS risk in periodontal disease and ascertain the no-CAD phenotype. **a** Strength of immune response to three epitopes (in *rows*) across study subjects (*n* = 96, in *lanes*), grouped by periodontal (H, G, or P) and CAD diagnosis (no-CAD, s-CAD, or ACS) shown as intensity plots. Relative abundance of immunoprofile features is shown in blue color-scale. These epitope biomarkers in a 3-biomarker generalized logistic model differentiate P group from H (Supplementary Fig. S11). *Vertical lanes* – subjects (*n* = 96); *rows* – epitopes; *blue color-scale –* normalized relative epitope-containing peptide abundance values (using 97.5^th^ percentile values per feature and capped at value 1). **b** Different immunoprofile features (*above boxplots*) stratified ACS with periodontal diagnosis. *y-axes*: immunoreactivity to epitopes in individual immunoprofiles, shown as abundance of peptides containing the epitope. Mann–Whitney *U* test, two-sided, *p* values not corrected for multiple comparisons, **p < 0.01, *p < 0.05. H: *n* = 21 independent patients; G: *n* = 27 independent patients; P: *n* = 48 independent patients. **c** Sensitivity and specificity measures of using biomarkers from B in separate periodontal groups (*sub-group*) to predict CAD diagnoses (either no-CAD or ACS). *Diagnosis* *≥* *threshold* – diagnosis group into which patient was classified if immune response was over threshold. **a**–**c** Abbreviations and group sizes: H -periodontally healthy (*n* = 21), G -gingivitis (*n* = 27), *P* -periodontitis (*n* = 48), *no-CAD* -no CAD diagnosis (*n* = 32), *s-CAD* - stable CAD (*n* = 32), *ACS - * acute coronary syndrome (*n* = 32). Data in File S7.

To examine whether the delineated five epitopes (A-E) could act as biomarkers stratifying periodontitis and/or CAD conditions, multi-variable models were built and fitted using antibody response to epitopes as predictive biomarkers. Firstly, it was confirmed that immune response to epitopes A-E had no significant correlations with age or gender (Supplementary Fig. S10). The optimal model including immune response to epitopes A, B, and C to differentiate periodontitis from healthy showed a balanced accuracy of 81% for the training subset (80% of samples) and 84% for the validation subset (20% of samples) (Fig. 5a and Supplementary Fig. S11). As a whole, strong response to epitopes A and B was characteristic to the periodontitis group, whereas response to epitope C was more common in the periodontally healthy group (Fig. 5a). Response to epitopes D and E did not provide additional useful information when discriminating between periodontitis diagnosis groups (Supplementary Fig. S12a).

Next, by characterizing immune features that were CAD-specific, we discovered that periodontally healthy subjects shared a strong response against epitope C that was high in ACS subgroup (with statistically significant trends, Mann–Whitney *U*, *p* = 0.097, two-sided, Fig. 5b). In subjects with gingivitis, a transient inflammation condition, response against epitope A was differentiating between CAD groups, being high in ACS as compared to no-CAD (Mann–Whitney *U*, *p < 0.05, two-sided, Fig. 5b). In the periodontitis group of subjects with chronic periodontal inflammation, antibody response to two independent markers (epitopes A and E) was identified as significant for the no-CAD cohort (Mann–Whitney *U*, *p < 0.05, **p < 0.01, two-sided, Fig. 5b). Response to epitopes B and D was not differentially linked to no-CAD or ACS diagnosis (Supplementary Fig. S12b). ROC analysis was used to set thresholds for each of the 3 epitopes (A, C and E) in discriminating between clinical subgroups (Fig. 5c). As a result, when first classifying subjects based on their periodontal findings (epitopes A, B and C) and then assessing response to epitopes A, C, and E which differentiate between CAD diagnoses, it was possible to predict the ACS risk in periodontal disease and ascertain the no-CAD phenotype (Fig. 5c). In conclusion, MVA immunoprofiling provided useful blood-biomarkers to predict ACS risk from periodontal disease.

## Discussion

Here, we report the detailed antibody epitope delineation study identifying a set of immunogenic features associated with periodontal pathogens and CAD.

The remarkable heterogeneity in antigenic immune response between individuals has been noted previously, also by our recent studies^30,31^. One of the factors shaping the individual heterogeneity of immune response is associated with microbial symbiosis with the host and their antagonistic to mutualistic associations^37^. Among oral bacteria, these include health-associated early-stage, moderately pathogenic medium-stage and highly pathogenic late-stage colonisers of periodontal biofilm^37,38^. Our data reveal that many strongly targeted epitopes could potentially mimic and the antibodies could cross-react with the antigens of periodontal bacteria (Fig. 2, Supplementary Table S2, Supplementary Fig. S4). Other factors influencing individual variability in immune response to pathogens are age, lifestyle (diet, smoking, exercise etc), previous immune history (viral, bacterial), and the specific HLA-alleles that affect the presentation of major antigenic epitopes^39^. As some of these periodontal bacteria belong to orange or red complex groups of pathogenic oral species, the observed potential cross-reactivity of the antibody immune response (Fig. 2, Supplementary Fig. S4) could contribute to the dysbiosis of oral microbiota and thereby periodontal health.

We identified immunoprofiles stratifying the individuals with ACS from individuals with no CAD or stable CAD (Fig. 3). Our dataset has the advantage that we could identify and compare the presence of potential immunological markers in different CAD types, even in a limited study cohort. When the host’s B cell response is inefficient against re-surfacing of latent infections, this may cause endothelial inflammation which in turn can contribute to the formation of atherosclerotic plaques and their instability^40^. Furthermore, differences were also observed in antibody response against epitopes in specific pathogens, including herpesviruses, in gingivitis, smoking or diabetes subgroups (Fig. 3, Supplementary Fig. S8). Diabetes along with smoking are two big risk factors for periodontal disease^41^. Thus, despite the small scale of the study and varied pathology background, our results provide proof of principle that different stages of CAD may be identifiable by different features of systemic immunoprofiles, which also include antibody response against highly antigenic epitopes of oral microbiota and common viruses.

Our data show that patients with progressing gingivitis through to periodontitis have increasing levels of antibodies to the highly antigenic epitope mimicking EBV VP26 (Figs. 3c and 4a, b). On the other hand, within subjects with periodontitis, a strong response to the EBV VP26 epitope is characteristic to healthy subjects, but not to ACS patients, suggesting its protective role against ACS (Fig. 5b). A few studies have directly addressed the role of herpesvirus infections in susceptibility to secondary oral bacterial infections. Relatedly, it is known that *A. actinomycetemcomitans* and *P. gingivalis* might require support from active herpesviruses for periodontal destruction, whereas the stable periodontal lesions may be devoid of viruses^42^. The herpesviral–bacterial hypothesis of periodontitis proposes that the herpesvirus infection triggers a release of proinflammatory cytokines to activate osteoclasts and matrix metalloproteinases (MMPs) to impair antibacterial immune mechanisms, causing an up-growth of periodontopathic bacteria^42^. Given that the extracellular matrix (ECM) breakdown represents a crucial factor in the periodontal pathophysiology, periodontitis confounded diagnostics of ACS has been proposed by measuring serum levels of MMP-9^43^, while serum MMP-8 and TIMP-1 levels were found to be associated with incident^44^ and especially fatal cardiovascular events^45^. Overall, this indicates immune hyperresponsiveness to dysbiosis, typical to the pathogenesis of periodontitis^46^. On the other hand, periodontal herpesviruses themselves may disseminate via the systemic circulation to non-oral sites (including arteries) and thus represent a major link between periodontitis and cardiovascular diseases^41^. A recent meta-report described a world-wide association of EBV infection with periodontal disease^47^. Here we have investigated this observation further and fine-mapped the EBV VP26 epitope, against which the immunologic response could link periodontitis and associated CAD conditions. However, herein we show that it mimics antigens of periodontal bacteria (Fig. 2d, Supplementary Table S2). Microbial epitope similarity with pathogens, allergens and auto-antigens has been reported before, as it can elicit tolerogenic or inflammatory immune reactivity^48^. For example, different periodontopathogenic species share mimicry in their GroEL antigen, which in turn results in cross-reactivity with the human heat-shock proteins (HSP) expressed on the endothelial cell leading to endothelial dysfunction^49^ and atherosclerosis^50^. This suggests that further studies of the herpesviral-bacterial-host epitope mimicry are warranted, in particular for improved diagnostics and therapy of dental health associated heart conditions.

Here we built a two-step biomarker model based on immune response to 4 epitopes that allows a) to classify subjects based on their periodontal diagnosis and b) to predict ACS risk and establish the no-CAD phenotype with 71–89% specificity (Fig. 5). Specific periodontitis-associated biomarkers for CAD could be beneficial in discerning ACS from other cardiac events. These data indicate that periodontitis, and ultimately putative progressing to ASC due to periodontitis, is the result of a partial response or lack of an efficient combined response against viral and bacterial infections landing on self-proteins.

Taken together, our findings clearly illustrate the power of MVA for the immunopathological analysis of oral health-related cardiac conditions, and we predict that the widespread use of this technology at scale will enhance the current understanding of chronic disease mechanisms, in particular cardiovascular diseases, and can lead to improved diagnostic accuracy and new markers.

### Limitations of the study

We could not evaluate the prognostic value of these predicted biomarkers due to the clinical study design. Because of genetic and socioeconomic status variabilities in different study populations, it is hard to extrapolate the findings. This was a study on Finnish adults. New studies with other cohorts are needed. Also, the pathogenetic importance of the specific oral bacterial microbiota antigens remains firmly to be established.

## Methods

### Ethics statement

The study was conducted in accordance with the guiding principles of the Declaration of Helsinki and the study participants gave written informed consent before enrollment. The study was approved by the ethics committees of the Helsinki University Central Hospital (approval reference number 106/2007) and The National Institute for Health Development, Estonia (approval number 1045).

### Clinical cohort description

The cohort (*n* = 96) was selected from the initial Corogene study (*n* = 5294) and divided into 3 subgroups: periodontally healthy (H, *n* = 21), gingivitis positive (G, *n* = 27), and periodontitis (P, *n* = 48). The diagnostic features on clinical and radiographic findings have been described in detail earlier^51,52^. Periodontally healthy patients had no alveolar bone loss (ABL) and bleeding on probing (BOP) did not exceed 10%. Gingivitis was registered in patients without ABL but with BOP > 10%. Patients were diagnosed with periodontitis when the ABL exceeded the cervical third of the root. Coronary artery disease diagnosis (no coronary artery disease (no-CAD, *n* = 32), stable coronary artery disease (stable-CAD, *n* = 32), or acute coronary syndrome (ACS, *n* = 32)) was based on the degree of stenosis in the coronary arteries during the angiography, typical electrocardiographic changes, chest pain, and levels of cardiac biomarkers^32^. The age and gender proportions, along with other relevant clinical history, are in Supplementary Table S1.

### Statistics and reproducibility

The statistical analyses performed during the study were accompanied by measures of statistical significance. The study was nonblinded and non-randomized and included *n* = 96 independent study subjects. Group-wise parameters, such as median values, were visualized alongside intra-group range using violin- or boxplots. Reproducibility of Mimotope Variation analysis was confirmed by establishing the correlation coefficient of two replicates as *R* = 0.87 (*p* < 0.0001).

### Statistical analysis of clinical characteristics of samples

Differences in proportions of genders, diabetes condition and smoking status were assessed in clinical sub-groups using Chi^2^ test (MedCalc, 19.7.2, MedCalc Software Ltd, Ostend, Belgium; https://www.medcalc.org). For statistical analysis, two-sided Mann–Whitney *U* test was used for comparing two groups or two-sided Kruskal–Wallis test for >2 groups using R package “ggpubr”^53^ and “ggplot2”^54^.

### Mimotope variation analysis

Peptide antigens were selected from random peptide phage modified library (PhD12, NEB) with 10^9^ different 12-mer peptide sequences^30,31^. Two μl of serum/plasma samples, previously precleared to plastic and E. coli/wt M13 phage lysates were incubated with 2.5 μl library (~5 × 10^11^ phage particles) and immunoglobulin G (IgG) fraction was recovered using protein G-coated magnetic beads (S1430S, NEB). Captured phage DNA was analyzed by Illumina HiSeq sequencing of 50-bp single end reads using barcoded primers for sample multiplexing. Peptide abundance correlation coefficient (R) in two replicates by Pearson analysis was 0.87 (*p* < 0.0001) (Supplementary Fig. S1) (R package “ggpubr”^53^). For further data analysis, sequencing errors and known artefacts were eliminated.

### Selecting peptides

Group-enriched peptides (TopPeptide sets) were selected for clinical sub-groups (Supplementary Table S1). Peptides were selected for no-CAD (*n* = 292,667), stable-CAD (*n* = 279,020), and ACS (*n* = 308,445), and periodontally healthy (*n* = 450,531 peptides), gingivitis (*n* = 450,590), and periodontitis (*n* = 342,261) groups, using the criteria that these were to be identified in ≥10% individual samples of the group with abundance threshold ≥10 sequence counts in at least one sample.

### Sequence-based unsupervised clustering of peptide antigens

Exhaustive sequence pattern search tool SPEXS2 was used (http://egonelbre.github.io/spexs2/) for sequence-based unsupervised clustering of peptide antigens. Starting from the 292,667 group-enriched peptides (TopPeptide set) identified for no-CAD group, all were used as input to SPEXS2 in random order with the search criteria: peptide coverage threshold: ≥4; motif coverage threshold: ≥4 fixed amino acid positions; hyper-geometric *P* value <10^−5^). Sequence pattern searches were performed in 2 iterative runs, where peptides from which a consensus was identified in the first run were excluded from the subsequent run. As a result, 4366 distinct motif consensus sequences were identified, which were contained in 29.0% or 84,873 of the original 292,667 peptides. Therefore, 4366 unique consensus motifs were identified for no-CAD (covering 29.0% of input peptides), similarly 2771 motifs were calculated for stable-CAD (27.0%), 4405 for ACS (30.5%), 6275 for periodontally healthy (4 iterative SPEXS2 runs due to greater starting peptide set) (42.8%), 9560 for gingivitis (4 runs) (44.4%), and 5936 for periodontitis group (4 runs) (27.7%) were defined.

### Selecting for group-differentiating motifs

The epitope motifs contained high degree of redundancy, therefore stricter criteria (hyper-geometric *P* value <10^−8^ or query/reference ratio ≥10) were imposed to select for characteristics with high significance and statistical power. Altogether 8088 unique motif sequences fit those criteria were selected for further analyses as the TopMotif set. Of 8088 distinct motif features, 995 were designated as group-differential. The 995 motifs satisfied all the criteria that 1) the average abundance value in each clinical sub-group was >3 greater than in another relevant sub-group, 2) the abundance-based separation of relevant clinical sub-groups was with ≥50% sensitivity and ≥70% specificity, and 3) group-separation was statistically significant (Mann–Whitney *U* test, *p* value < 0.05, two-sided).

### Alignment profiles on periodontal bacteria

The TopMotif set of 8088 motifs was aligned to proteomes of the periodontal pathogen species: *Porphyromonas gingivalis* (UniProt accession: UP000000588), *Tannerella forsythia* (UP000005436), *Prevotella intermedia* (UP000010099), *Fusobacterium nucleatum* (UP000002521), *Campylobacter rectus* (UP000003082), *Aggregatibacter actinomycetemcomitans* (UP000002569), and *Porphyromonas endodontalis* (UP000004295) (accessed 20-21.12.2019). Only exact alignments where all fixed amino acid positions of a motif (minimum of 4 positions) to match with the target were allowed. The pathogen database comprised of 15,928 proteins, of which 15,116 matched at least with one epitope motif. For each pathogen, the individual alignment load was calculated per 20-amino acid fragment of the protein, in two frameshifts (0 and −10 aa), yielding in 10 aa overlaps between considered fragments. Altogether we analyzed ~480,000 distinct 20 amino acid fragments. The top 40 fragments for each bacterial species, from any frameshifts, with the highest total alignment loads were selected based on the formula:

$${Alignment}\,{load}\,({per}\,20{aa}\,{fragment})=\,\frac{{sum}\,{of}\,{abundance}\,{of}\,{motifs}\,{aligned}}{{count}\,{of}\,{motifs}\,{aligned}}$$

If higher alignment loads were encompassing two side-by-side regions (one with a frameshift), a longer 30-amino acid fragment was considered. Individual alignment loads (Supplementary Data 1) were compared across pathogen species using Kruskal–Wallis test for comparing all groups and two-sided Mann–Whitney *U* test for pair-wise comparisons. R packages “ggpubr” and “ggplot2” were used for calculation and visualization.

### Identifying target types of potential epitopes of periodontal bacteria

Using the top 40 antigen fragments of periodontal pathogens, 12mer substrings were extracted for each aligned epitope motif (of the TopMotif set), whereas substrings shorter than 12mer were discarded (33 were found). Altogether 1691 substrings were selected, position weight matrices were built and the resulting enriched amino acid positions were visualized as sequence logos, all using a custom in-house tool with WebLogo^55,56^ integration (parameters: no counts, distance cut-off 9, 10 minimum unique substrings in a cluster, similarity index 15). The analysis yielded 26 target types with consensus sequences, of which top 5 most prevalent are shown on Fig. 2b.

### Amino acid-based clustering of group-differential epitopes

Sequence homology clustering analysis reduced 705 out of 995 epitope motifs into 62 clusters of similar epitopes (≥3 identical amino acid positions) whereas 290 out of 995 epitope motifs either formed too small clusters (<3 motifs in a cluster) or were not similar enough with any other epitope (<3 identical amino acid positions). Additional R packages used in data analysis and visualization were: “readr”, “dplyr”^57^.

Based on the selected peptides, position weight matrices were built and the resulting enriched amino acid positions were visualized as sequence logos, all using a custom in-house tool with WebLogo^55,56^ integration.

### Group-specific analysis of 62 clusters

The average abundance for each of the 62 clusters with the defined sequence logo was calculated across clinical sub-groups. Abundance values in clinical sub-groups were normalized with average abundance across all groups (Supplementary Data 2). Clusters with average abundance values of <150 were left out of further analysis. Next, hierarchical clustering was performed based on the normalized abundance values using Pearson correlation coefficients for clustering distance and ward.D2 clustering method (R package “pheatmap”)^58^. R packages used in data analysis and visualization included “readr”, “dplyr”, “forcats”, “reshape2”^54,57^, and “viridis”^59^.

### Individual-based clustering of 62 clusters

Abundance of peptides in individual immunoprofiles was calculated for each cluster. Using log10 values of the abundances, Pearson correlation coefficients R were calculated pair-wise for all 62 clusters (Supplementary Data 3). The clusters were subsequently grouped using R package “pheatmap” with ward.D2 as clustering method and Pearson correlation coefficients as clustering distance. Five distinct larger groups were identified and defined as epitopes A-E. For each of the epitopes A-E, the containing clusters were compared to determine consensus sequences describing the core epitopes (Supplementary Data 7).

### Alignment on EBV VP26

Epitope A has been previously mapped to Epstein-Barr virus (EBV) protein VP26^30^. To validate the mapping in the current clinical cohort, epitopes from individual immunoprofiles (*n* = 96) were aligned to the primary sequence of EBV protein VP26, a component of the viral capsid antigen (VCA) (UniProt accession code Q3KSU9, EBV strain GD-1, date accessed: 25.11.2020). Signal-to-random ratio for each sample was calculated across primary sequence of EBV VP26, where signal represented the count of aligned (≥6 matching amino acid positions) unique peptides defined by MVA and random represented the count of aligned random peptides (≥6 matching), generated by sequence scrambling of peptides (Supplementary Data 4). As controls (Ctrl), peptides from EBV CA seronegative subjects (*n* = 9) were analyzed similarly.

### ELISA

CMV and EBV serostatus was measured from serum samples with ISO/IEC 17025 accredited methods. In brief, serological analyses were performed with anti-CMV IgG ELISA method (EUROIMMUN EI 2570-9601G) and with anti-EBV CA (capsid antigen) IgG ELISA method (EUROIMMUN EI 2791-9601G) according to manufacturer’s specifications. Absorbance was measured at 450 nm with SpectraMax Paradigm (Molecular Devices).

### Dot ELISA

MVA analysis findings were validated by dot ELISA analysis. For that, M13K phages displaying peptides with either the EBV VP26 epitope-containing sequence TLPMDTSPRAHW or the mutant sequence TLPMDASPRAHW as parts of the pIII protein were printed onto nitrocellulose (NC) slides (ArrayIt, US). Unspecific binding was reduced by blocking for 1 h at room temperature with 5% non-fat dried milk in 1xPBS-Tween20-0.05%. Human serum samples were precleared to reduce unspecific binding to M13K phages, to *E. coli* bacterial proteins and to plastic. Preclearing step was performed in mix of 60 µl 2.5% skimmed milk-1xPBS-0.05% Tween20 + 30 µl Preabsorption Solution + 1:50 serum, at 4 °C overnight. Following this, the slides were incubated with either precleared serum 1:50 solutions (*n* = 54) or with 1:5000 mouse anti-M13 antibody (#27-9420-01, GE healthcare) in 2.5% skimmed milk in 1xPBS-0.05% Tween20 (GE Healthcare) for 1 h at room temperature. The anti-M13K antibodies were used to quantify phages printed on NC slides. Multiple washes were performed with 5% skimmed milk in 1xPBS-0.05% Tween20. For visualization, the secondary antibodies used were 1:1000 rabbit anti-human HRP-conjugated antibody (#ab6759, Abcam) (for human serum samples) in 2.5% skimmed milk in 1xPBS-0.05% Tween20 or 1:1000 rabbit anti-mouse HRP-conjugated antibody (#ab6728, Abcam) (for anti-M13K) in 2.5% skimmed milk in 1xPBS-0.05% Tween20, incubated for 1 h at room temperature. After multiple washes, the presence of bound human sera/plasma IgG antibodies was detected via reaction with HRP substrate DAB chromogen diluted in substrate buffer (1:100). The slides were digitally scanned and the signals were quantified using ImageQuantTL (version 8.1) (Supplementary Data 5). Results of 27/54 samples were validated in an independent similar experiment (in total *n* = 2 experiments) (including all samples which showed high signal-to-background ratios).

### Predicting periodontitis and CAD diagnoses

Generalized linear model was fit to 80% of subjects’ data (with 5x cross-validation) to classify different case-subgroups based on their immunoprofile features. Using model’s prediction probabilities for subjects in the training set, receiver operating characteristic (ROC) analysis was performed. The model’s area under the receiver operating characteristic curve (AUROC) was 0.843 with 95% CI (0.738…0.948). The model was validated on the validation subset (20% of samples).

### Data visualization

Box and whisker plots were generated in the style of Tukey with R packages “ggpubr” or “ggplot2”. Upper, middle and lower boxplot lines represent the 75th, 50th and 25th percentiles, while whiskers represent the largest or smallest value within 1.5 times interquartile range above the 75th percentile or below the 25th percentile, respectively. Individual data points without outliers are visualized.

### Reporting summary

Further information on research design is available in the Nature Research Reporting Summary linked to this article.

## Supplementary information

Supplementary Information

Description of Additional Supplementary Files

Supplementary Data 1

Supplementary Data 2

Supplementary Data 3

Supplementary Data 4

Supplementary Data 5

Supplementary Data 6

Supplementary Data 7

Reporting Summary

## Data Availability

The source data analyzed during the study was generated by MVA analysis. We provided the relevant data underlying the main findings in the Supplementary data. The whole datasets generated and/or analyzed during the current study are not publicly available due to containing sensitive clinical information but are available from the corresponding author on reasonable request.

## Code availability

The code used during the current study is not publicly available due to its proprietary nature, but detailed explanation of the analysis approaches is available from the corresponding author on reasonable request.
