# Supplementary material for: Antibody response to oral biofilm is a biomarker for acute coronary syndrome in periodontal disease
Source: Commun Biol. 2022 Mar 4;5:205. doi: 10.1038/s42003-022-03122-4 (PMC8897497; doi:10.1038/s42003-022-03122-4)
Supplement: Supplementary file 1 — Supplementary Information [file 42003_2022_3122_MOESM1_ESM.pdf]

## Supplementary Information

**Table S1. Characterization of clinical groups, refers to Figure 1.** Based on periodontal health, the study cohort was divided into 3 subgroups: periodontally healthy (H), gingivitis (G), or periodontitis (P). Coronary artery health – no coronary artery disease (no-CAD), stable coronary artery disease (stable-CAD), or acute coronary artery disease (ACS) - was based on the degree of stenosis in the coronary arteries during the angiography, typical electrocardiographic changes, chest pain, and levels of cardiac biomarkers. For all of the clinical diagnosis groups, mean age ± SD, gender proportions, mean body mass indices (BMI), and proportions of diabetic subjects or cigarette smoking subjects were calculated. $Chi^2$ test was performed to assess proportional differences of diabetes or smoking behaviour groups across CAD or periodontitis groups.

| **Diagnosis group** | **no-CAD** | **stable-CAD** | **ACS** | N | Age (mean) ± SD, years | Gender (M/F) | BMI (mean) | Diabetes (No/Yes) | Smoking (Never/Ex-smoker/Yes) |
|---|---|---|---|---|---|---|---|---|---|
| **H** | *7* | *7* | *7* | 21 | 60.0 ±7.9 | 14 M/ 7 F | 28.4 | 18 No/ 3 Yes | 12 Never/ 8 Ex-smoker/ 1 Yes |
| **G** | *9* | *9* | *9* | 27 | 60.5 ±9.6 | 17 M/ 10 F | 29.9 | 21 No/ 5 Yes | 21 Never/ 5 Ex-smoker/ 1 Yes |
| **P** | *16* | *16* | *16* | 48 | 63.6 ±7.7 | 36 M/ 12 F | 27.9 | 38 No/ 10 Yes | 16 Never/ 20 Ex-smoker/ 12 Yes * |
| N | 32 | 32 | 32 | | | | | | |
| Age (mean) ± SD, years | 60.2 ±8.3 | 64.3 ±8.4 | 61.3 ±8.2 | | | | | | |
| Gender (M/F) | 17 M/ 15 F | 26 M/ 6 F | 24 M/ 8 F | | | | | | |
| BMI (mean) | 27.9 | 28.4 | 29.4 | | | | | | |
| Diabetes (No/Yes) | 29 No/ 3 Yes | 23 No/ 8 Yes | 25 No/ 7 Yes | | | | | | |
| Smoking (Never/Ex-smoker /Yes) | 17 Never/ 10 Ex-smoker/ 5 Yes | 16 Never/ 13 Ex-smoker/ 3 Yes | 16 Never/ 10 Ex-smoker/ 6 Yes | | | | | | |

* $Chi^2$ test, $p$-value < 0.01

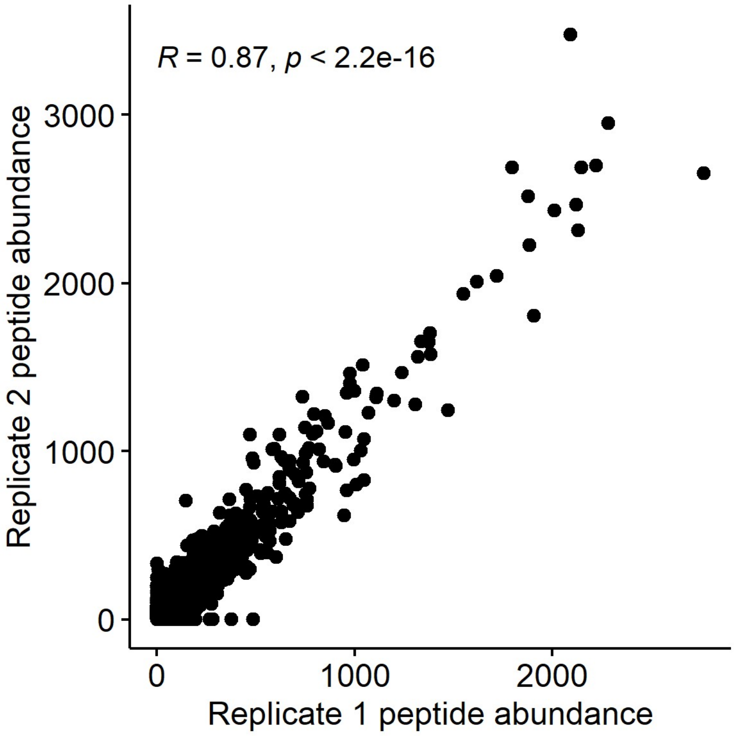


**Figure S1. Pearson correlation of R=0.87 between MVA peptide antigen profiles of two independent replicates confirms reproducibility of MVA analyses, refers to Mimotope Variation Analysis in Methods.** R programming language and package “ggpubr” was used to calculate correlation and perform hypothesis test on the correlation coefficient (p < 2.2e-16), and together with package “ggplot2” was used to visualize the results [35, 36].

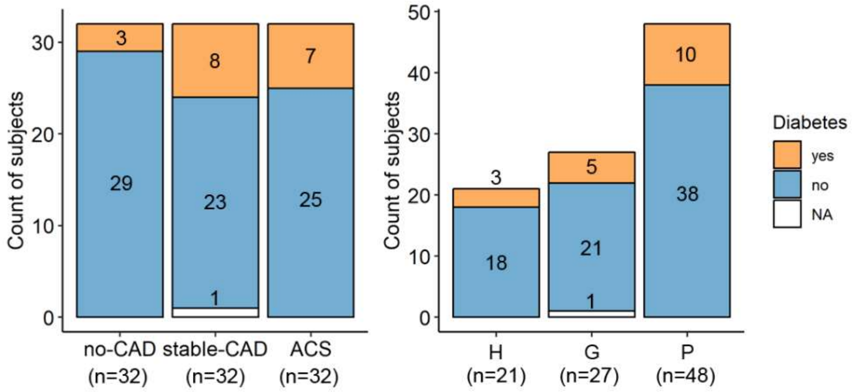


**Figure S2. Although 19% of the subjects were diagnosed with diabetes (either type I or type II, not specified further), no significant association of diabetes with either CAD or periodontal diagnosis was found, refers to Figure 1.** Frequency distribution graphs of 96 individuals divided into groups by coronary artery health condition (no-CAD, stable-CAD, or ACS) or periodontal condition (periodontally healthy (H), gingivitis (G), periodontitis (P)). No significant difference in diabetes diagnosis prevalence was observed across coronary artery health or gum inflammation condition groups. The potential association of groups was assessed using Chi$^2$ test, statistical significance denoted from p-value $< 0.05$. *x-axes – clinical subgroup distinctions; y-axes – count of subjects; color-fill – yes: diagnosis of diabetes (either type I or II) (orange), no: no diabetes diagnosis (blue), NA: one subject unknown (white).*

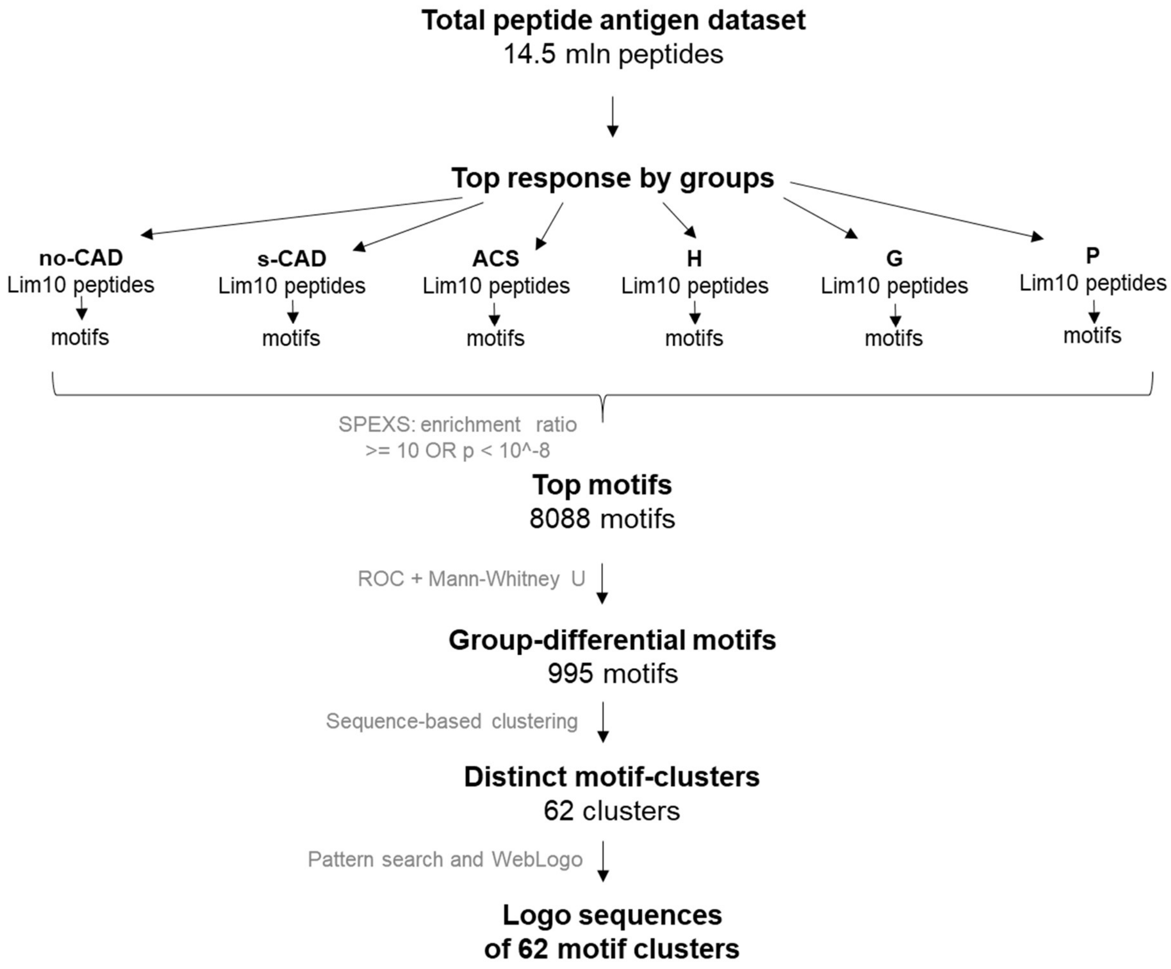


**Figure S3. Data analysis workflow from MVA-delineated peptide antigens to motif clusters differentiating clinical groups, related to data analysis in Methods.** The MVA experiment concluded 14.5 million distinct peptide sequences for the cohort of 96 subjects. Divided into clinical subgroups (no-CAD, s-CAD, ACS or H, G, P), most abundant and shared peptides were extracted as TopResponse peptide sets (abundance value >= 10 and detected in >= 10% samples). Exhaustive pattern detection analysis was performed using SPEXS2 algorithm iteratively (and scrambled peptide set as random), resulting in shorter consensus motif sequences (http://egonelbre.github.io/spexs2/). Of those, 8088 were selected by enrichment parameters (compared to the used random reference) as TopMotifs set (enrichment ratio >= 10 or hypergeometric p-value < 10^-8). Based on the abundance of motifs in individual samples, 995 were identified as group-differential (either across CAD or oral health groups), using Receiver Operating Characteristic (ROC) analysis and Kruskal-Wallis statistical tests. Amino acid sequence-based clustering of the 995 motifs resulted in 62 motif clusters (containing 705 motifs). Custom pattern search tool together with WebLogo tool were used for generating logo (amino acid enrichment) sequences for each of the 62 motif clusters.

**Table S2. Common epitope patterns among top 40 antigen fragment sequences, refers to Figure 2.** Under each bacterial species there is a Uniprot accession code referencing to the protein and the number range represents the amino acid position range of the antigen fragment.

| **Type 1: KP.L (85 antigen fragments)** | **Type 2: P..T.[P]R (54 antigen fragments)** | **Type 3: N[ST]F.K (35 antigen fragments)** | **Type 4: P[AYS][LI]TA.[REQ]-[GT][LDK] (12 antigen fragments)** | **Type 5: PQ[DN]T[RIV]P[MIR][GRT][MRK] (8 antigen fragments)** |
|---|---|---|---|---|
| P. gingivalis | P. gingivalis | P. gingivalis | P. gingivalis | P. gingivalis |
| *Q7MXW4_100-120* | *Q7MXF5_50-69* | *Q7MWV7_0-20* | *Q7MX90_240-269* | *Q7MVY1_440-460* |
| *Q7MXU3_110-129* | *Q7MX79_150-169* | *Q7MV39_340-369* | A. actinomycetemcomitans | *Q7MTA5_220-240* |
| *Q7MXP7_70-89* | *Q7MX79_160-189* | F. nucleatum | *C9R6F4_200-220* | A. actinomycetemcomitans |
| *Q7MWT1_160-180* | *Q7MWU8_440-460* | *Q8RIS2_90-109* | *C9R664_200-229* | *C9R256_70-89* |
| *Q7MVZ8_330-349* | *Q7MWP7_480-500* | *Q8RIQ9_240-260* | *C9R5T4_370-389* | F. nucleatum |
| *Q7MVB8_50-69* | *Q7MVZ7_260-280* | *Q8RIM5_110-129* | *C9R5E4_90-109* | *Q8REZ1_140-160* |
| *Q7MV13_20-49* | *Q7MVX8_900-920* | *Q8RHZ5_420-440* | *C9R464_100-120* | P. endodontalis |
| *Q7MTU0_180-200* | *Q7MV60_120-140* | *Q8RHG1_60-80* | F. nucleatum | *C3JAY9_440-460* |
| *Q7MTQ7_70-89* | *Q7MUF8_100-120* | *Q8RG91_800-820* | *Q8RI28_80-100* | T. forsythia |
| A. actinomycetemcomitans | *Q7MTZ6_350-369* | *Q8RFZ6_240-260* | P. endodontalis | *G8UQL3_20-49* |
| *D0UIW1_510-529* | *Q7MTV2_490-509* | *Q8RFV3_1430-1449* | *C3JAW0_30-49* | *G8UIT4_190-209* |
| *D0UIQ1_350-369* | *Q7MTG1_100-129* | *Q8RFF6_600-620* | T. forsythia | *G8UHL5_270-289* |
| *C9R7G3_190-209* | *Q7MT54_0-20* | *Q8RFC1_390-409* | *G8UQE1_110-129* | |
| *C9R7E3_280-300* | A. actinomycetemcomitans | *Q8REF7_640-660* | *G8UJD6_40-69* | |
| *C9R6W6_330-349* | *C9R644_770-789* | *Q8RDZ9_240-260* | P. intermedia | |
| *C9R6U2_70-89* | *C9R5B4_420-440* | *Q8RDV0_120-140* | *I1YWW3_280-300* | |
| *C9R6N8_660-680* | *C9R3I3_20-40* | *Q8R6I3_240-260* | C. rectus | |
| *C9R5B3_240-260* | *C9R2A7_60-80* | *Q8R623_460-480* | *B9D2B9_280-300* | |
| *C9R4U7_580-609* | *C9R1V0_370-389* | *Q8R5W4_80-100* | | |
| *C9R414_440-460* | F. nucleatum | P. endodontalis | | |
| *C9R3P4_440-460* | *Q8RHC8_270-289* | *C3JD08_1-29* | | |
| *C9R3K6_580-600* | P. endodontalis | *C3JBV4_120-140* | | |
| *C9R2W4_520-549* | *C3JD67_300-320* | *C3JBD8_230-249* | | |
| *C9R2K1_260-280* | *C3JCV9_360-380* | T. forsythia | | |
| *C9R2H8_200-220* | *C3JCS1_150-169* | *G8UR08_80-100* | | |
| *C9R2E6_0-20* | *C3JCJ1_110-129* | *G8UJJ8_140-160* | | |
| *C9R2C5_290-309* | *C3JCB6_400-420* | *G8UHY0_920-949* | | |
| *C9R1V8_120-140* | *C3JCA3_120-140* | P. intermedia | | |
| *C9R1P1_100-120* | *C3JBY6_160-180* | *I1YWY8_120-140* | | |
| *C9R1J3_230-249* | *C3JBV7_210-229* | *I1YWT6_0-20* | | |
| F. nucleatum | *C3JBQ2_120-149* | *I1YW99_140-169* | | |
| *Q8RI66_380-400* | *C3JB08_390-409* | *I1YTF0_300-320* | | |
| *Q8RI07_80-100* | *C3JAZ1_1-29* | *I1YT31_70-89* | | |
| *Q8RGD3_440-460* | *C3JAQ0_370-389* | *I1YSZ4_310-329* | | |
| *Q8RFV2_310-329* | *C3JA63_280-300* | C. rectus | | |
| *Q8RF16_70-89* | T. forsythia | *B9D5M3_130-149* | | |
| *Q8REW5_80-100* | *G8UQ56_420-440* | *B9D3M2_0-20* | | |

| | | | | |
|---|---|---|---|---|
| *Q8REA9_0-20* | *G8UNM4_40-60* | *B9D204_340-360* | | |
| *Q8RE30_280-300* | *G8UN46_480-500* | *B9D004_60-89* | | |
| *Q8R6B0_70-89* | *G8UMK7_160-189* | *B9CYG9_160-180* | | |
| P. endodontalis | *G8UL86_880-900* | | | |
| *C3JCV9_410-429* | *G8UK79_290-309* | | | |
| *C3JCV9_420-440* | *G8UJQ1_160-180* | | | |
| *C3JCD9_210-229* | *G8UJ64_620-640* | | | |
| *C3JC37_620-640* | *G8UIT1_70-89* | | | |
| *C3JBZ0_170-189* | *G8UIL9_1020-1049* | | | |
| *C3JBC9_140-160* | P. intermedia | | | |
| *C3JA68_200-220* | *I1YWS8_330-349* | | | |
| *C3JA61_10-29* | *I1YUP8_340-360* | | | |
| T. forsythia | *I1YUA3_140-160* | | | |
| *G8UPY1_340-369* | *I1YU83_20-40* | | | |
| *G8UN54_330-349* | *I1YTW3_100-120* | | | |
| *G8ULU5_70-89* | *I1YS41_160-180* | | | |
| *G8ULU0_420-440* | C. rectus | | | |
| *G8UL95_260-280* | *B9D5R9_190-209* | | | |
| *G8UKL6_350-369* | *B9D4Y0_220-240* | | | |
| *G8UKK1_200-229* | *B9D3W2_680-700* | | | |
| *G8UJ34_530-549* | *B9D120_0-20* | | | |
| *G8UIN6_320-340* | *B9CZB9_20-40* | | | |
| *G8UHV7_260-280* | *B9CXY2_410-429* | | | |
| P. intermedia | | | | |
| *I1YWQ2_360-389* | | | | |
| *I1YWI7_0-20* | | | | |
| *I1YVZ8_1060-1080* | | | | |
| *I1YUS6_70-89* | | | | |
| *I1YUL4_720-749* | | | | |
| *I1YUI1_360-380* | | | | |
| *I1YUF5_480-500* | | | | |
| *I1YUB7_820-840* | | | | |
| *I1YU27_1200-1220* | | | | |
| *I1YTT2_360-389* | | | | |
| *I1YSY9_60-80* | | | | |
| *I1YSX6_60-80* | | | | |
| *I1YSC7_290-309* | | | | |
| *I1YS95_10-29* | | | | |
| *I1YS76_100-120* | | | | |
| *I1YRX4_130-149* | | | | |
| C. rectus | | | | |
| *B9D533_140-169* | | | | |
| *B9D428_520-540* | | | | |
| *B9D3Y7_500-520* | | | | |
| *B9D3W9_100-120* | | | | |

| | | | | |
|---|---|---|---|---|
| *B9D349_280-300* | | | | |
| *B9D2Z3_80-109* | | | | |
| *B9D2V6_290-309* | | | | |
| *B9D2G3_80-100* | | | | |
| *B9D1J1_240-269* | | | | |
| *B9D157_160-180* | | | | |
| *B9D008_230-249* | | | | |
| *B9CZA0_70-89* | | | | |
| *B9CY28_60-80* | | | | |

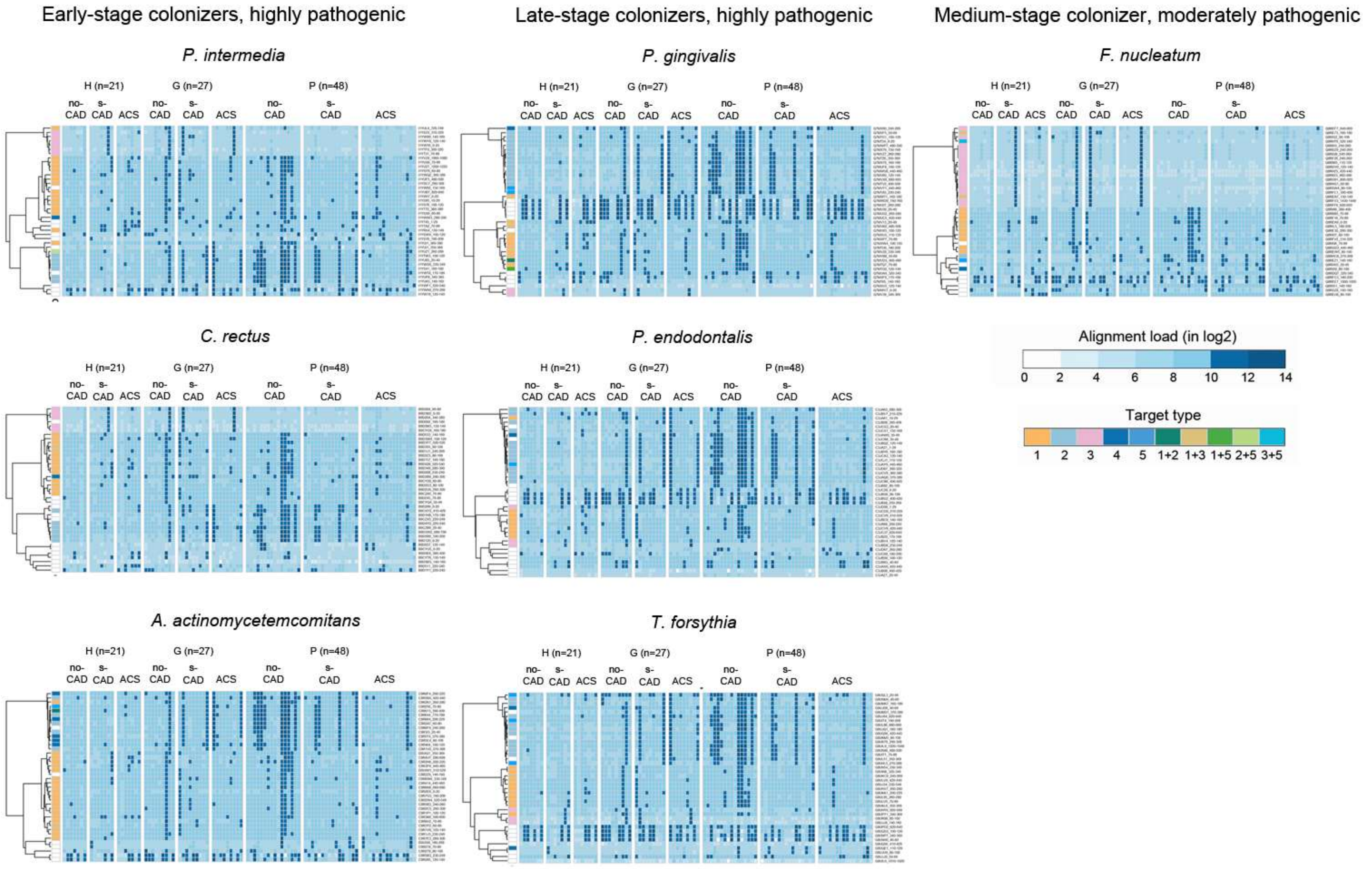


**Figure S4. Periodontal pathogens have potential antigenic epitopes targeted by top immune response, refers to Figure 2.** Group-wide top 8088 motif sequences were aligned onto proteomes of periodontal pathogens: *P. gingivalis, Porphyromonas endodontalis,*

*Tannerella forsythia, Prevotella intermedia, Campylobacter rectus, Aggregatibacter actinomycetemcomitans*, and *Fusobacterium nucleatum*. Alignment load values were calculated per 20-amino acid protein fragments for individuals separately. Top 40 fragments with highest alignment loads were visualized per pathogen. *Vertical lanes* – individual samples (n=96), categorized based on CAD (no-CAD, s-CAD, ACS) and periodontal diagnoses (H, G, P); *rows* – each row represents a distinct 20-aa region of protein primary sequence; *blue color-scale* – intensity of blue represents the alignment load of individual sample; *target type* – consensus sequence type of the 20-aa fragment (sequences in **Figure 2c**).

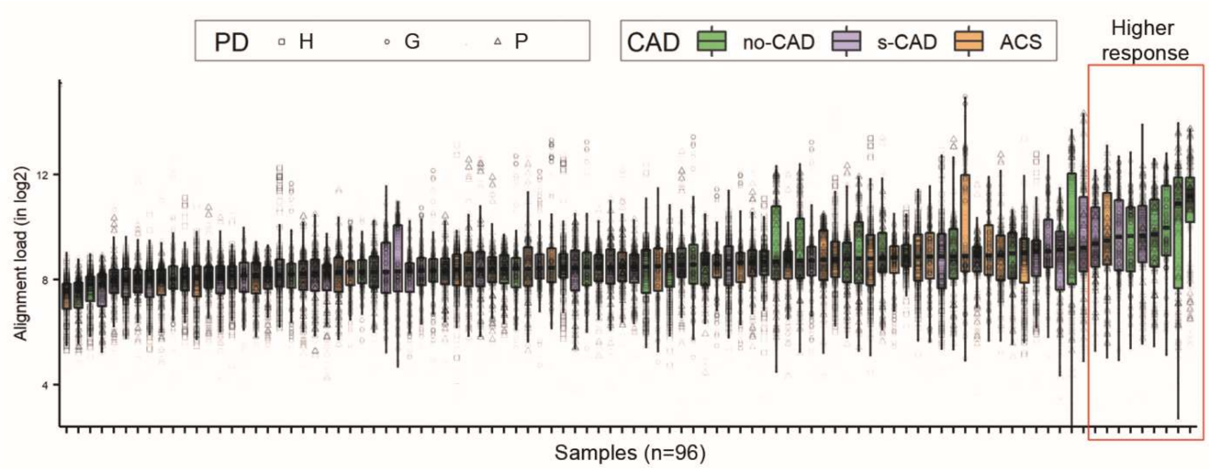


**Figure S5. Individual peptide alignment loads to antigens of periodontal bacteria across study cohort, refers to Figure 2.** The alignment loads (n=96) for the top 40 protein fragments of periodontal bacteria were compared between samples. Outlined are 9 subjects with high seroreactvity to select periodontal antigens, determined by upwards kink/elbow in the sorted median values. Clinical diagnoses of subjects are shown both in color (CAD diagnosis) and shape of data points (periodontitis diagnosis (PD)). Kruskal-Wallis test, **** p<0.0001. *x-axis* - 96 subjects; *y-axis* – alignment loads (in log2).

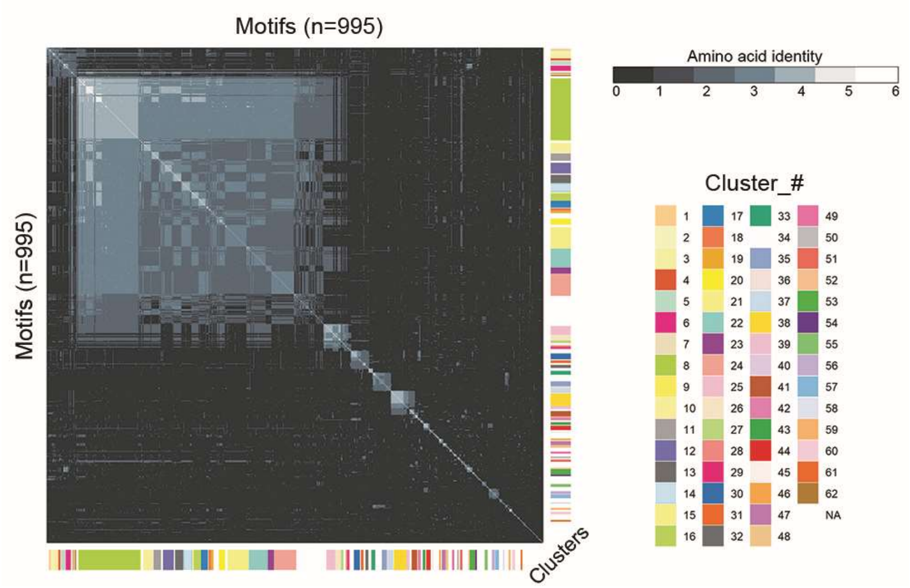


**Figure S6. Amino acid sequence-based clustering revealed 62 distinct epitope clusters with shared core patterns, refers to Figure 3.** The 62 epitope clusters are designated with colors above and next to the matrix. Amino acid identity measure between motifs (995 motifs shown) is color-coded in gray, ranging from 0 (least similar) to 6 (most similar). Motifs not included in any clusters are designated with *white space* above the matrix and denoted as "NA".

**Table S3. Consensus sequences of 62 peptide antigen clusters, refers to Figure 3.** The clusters numbered 1-62 were defined in **Figure S6** by amino acid sequence similarity. Based on the motif amino acid sequences within each cluster, common consensus sequences were determined. Based on the similarity of abundance levels (Pearson correlation) across individual samples (n=96), some of the 62 clusters were further grouped and defined into major epitopes A-E (**Figure 3a-b**). Clust_45 was discarded for low abundance in immunoprofiles, having <50 distinct peptide antigens which included any of the clust_45 motif sequences.

| Cluster | Consensus sequence | Epitope identification | | Cluster | Consensus sequence | Epitope identification |
|---|---|---|---|---|---|---|
| clust_1 | W[S]PF[S] | C | | clust_38 | PY..YQ | B |
| clust_2 | SPF | C | | clust_39 | PY..Y | B |
| clust_3 | T.P.SP[FL] | C | | clust_40 | [A]P...YQ | B |
| clust_4 | P..PF[TLI] | C | | clust_41 | P[ST].FY | |
| clust_5 | PW.P | C | | clust_42 | Y.HPS | |
| clust_6 | PD.P[IVN] | | | clust_43 | Y.[PS]TL.Y | |
| clust_7 | P..PQ.T.PR | A | | clust_44 | HHAP | |
| clust_8_1 | P..T.PR | A | | clust_46 | L..[P]...SSK | |
| clust_8_2 | LPWS[P]V | C | | clust_47 | MGG.K | |
| clust_9 | P.DT.P[R]S | A | | clust_48 | TG.TS | |
| clust_10 | PQ.T.P | A | | clust_49 | P..LL[TY] | |
| clust_11 | P.[SD]T.P | A | | clust_50 | LP[H]W[VT] | |
| clust_12 | PQ.T.PR | A | | clust_51 | L.WP.[P]K | |
| clust_13 | PQ.T.[P]R | A | | clust_52 | R.PT.[F].N | |
| clust_14 | DT.PR | A | | clust_53 | PD.[PA][VI]S | |
| clust_15 | TSPR | A | | clust_54 | K[T].TVL | |
| clust_16 | P.DT.[P]R | A | | clust_55 | DH.R | |
| clust_17 | P.DT.PR | A | | clust_56 | AHH | |
| clust_18 | P..[T]SPR | A | | clust_57 | A.HE | |
| clust_19 | P.S[T].PR | A | | clust_58 | V.E...ND.K | |
| clust_20 | P..TS[P]R | A | | clust_59_1 | SF[T]K | D |
| clust_21 | T.PR | A | | clust_59_2 | YIN.F | D |
| clust_22_1 | P..T..R | A | | clust_60 | GSVR | |
| clust_22_2 | SP....PR | A | | clust_61 | L.A..TG | |
| clust_23 | P..T.P | A | | clust_62 | PQ.PY | |
| clust_24 | P....PR | A | | | | |
| clust_25 | P.HT.K | E | | | | |
| clust_26 | GP.[H]T.K | E | | | | |
| clust_27 | P.HT.K | E | | | | |
| clust_28 | GP.HT.K | E | | | | |
| clust_29 | GP..[T]NK | | | | | |
| clust_30 | KP.LG | | | | | |
| clust_31 | KP.L | | | | | |
| clust_32 | PE.KP | | | | | |
| clust_33 | LK.H.K | | | | | |

| | | | | | | |
|---|---|---|---|---|---|---|
| clust_34 | NSF.K | D | | | | |
| clust_35 | N.F.K | D | | | | |
| clust_36 | AY.N...K | D | | | | |
| clust_37_1 | N[TDS]M.K | D | | | | |
| clust_37_2 | NT..K | D | | | | |

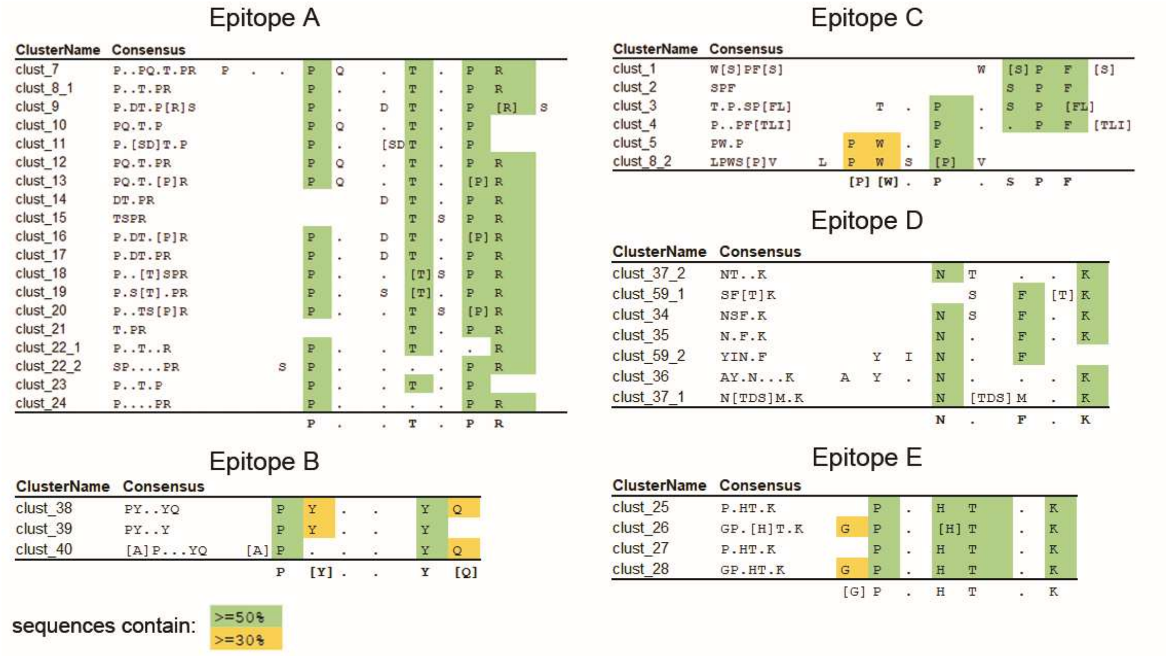


**Figure S7. Determining consensus sequences of correlating clusters, refers to Figure 3.** Epitope A-E refer to groups of correlating clusters from **Figure 3a**. ClusterName refers to clusters from the 62 clusters set, shown here with their consensus sequences. Comparisons of these consensus sequences in turn identified shared amino acid positions (green – shared between ≥50% clusters, yellow – shared between ≥30% clusters).

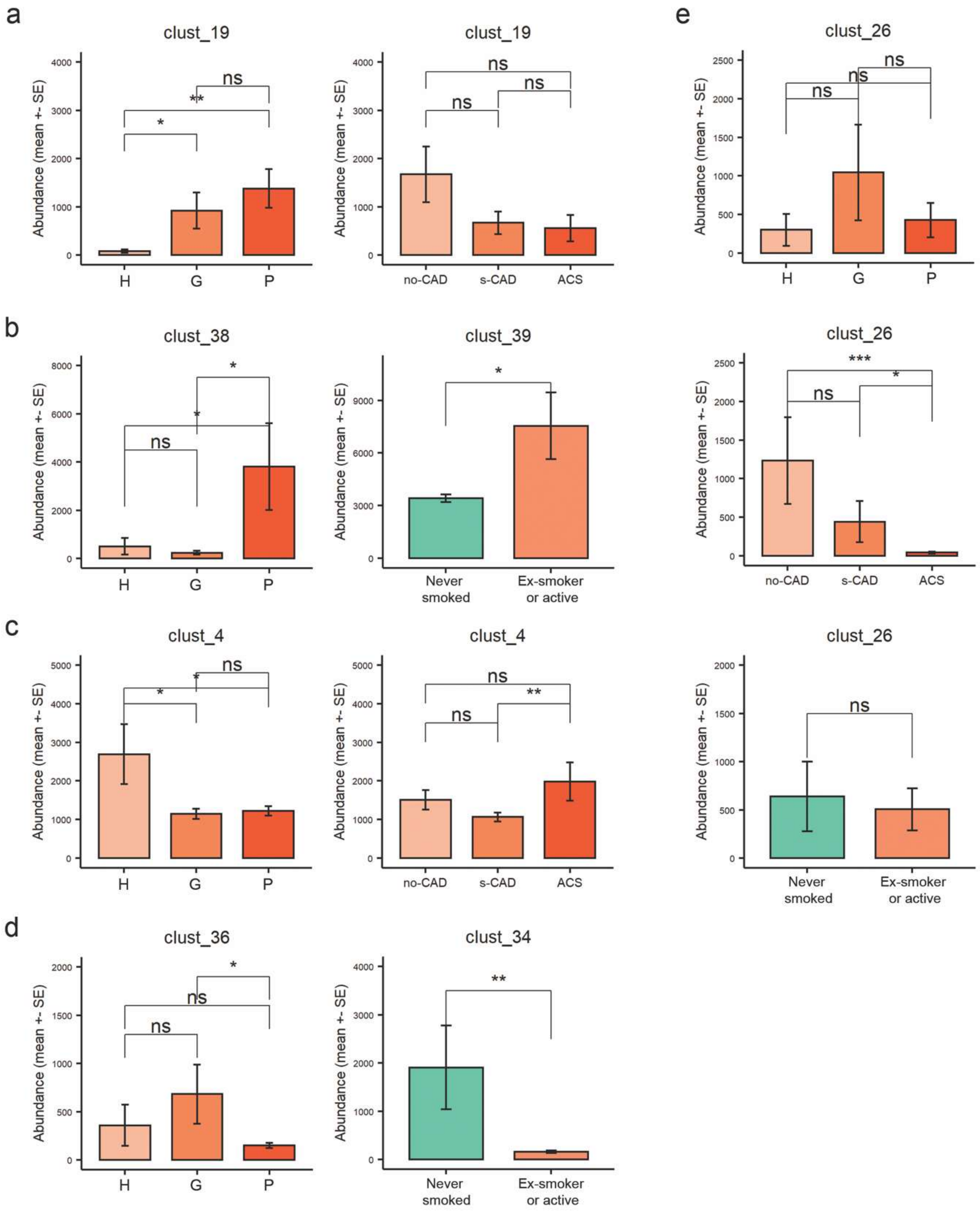


**Figure S8. Abundance of peptides containing consensus sequences of the specific peptide antigen clusters, refers to Figure 3.** Bar graphs represent mean abundance of the peptide antigens in a given clinical diagnosis group, error bars represent standard error. Group sizes and abbreviations: H – periodontally healthy patients (n=21); G – patients with gingivitis (n=27); P – patients with periodontitis (n=48); no-CAD – patients without coronary artery disease (CAD) diagnosis (n=32); s-CAD – patients with stable CAD diagnosis (n=32); ACS – patients with acute coronary syndrome (n=32); Never smoked – patients who have never smoked cigarettes (n=); Ex-smoker or active – patients who have previously smoked or are currently actively smoking (n=). Mann-Whitney U test, p-values not adjusted for multiple comparisons, ns p>0.05, * p<0.05, ** p<0.01. **a.**

Abundance of peptides containing epitope A cluster consensus sequences across periodontal health or CAD diagnosis groups. **b.** Abundance of peptides containing epitope B cluster consensus sequences across periodontal health or cigarette smoking groups. **c.** Abundance of peptides containing epitope C cluster consensus sequences across periodontal health or CAD diagnosis groups. **d.** Abundance of peptides containing epitope D cluster consensus sequences across periodontal health or cigarette smoking groups. **e.** Abundance of peptides containing epitope E cluster consensus sequences across periodontal health, CAD diagnosis, or cigarette smoking groups.

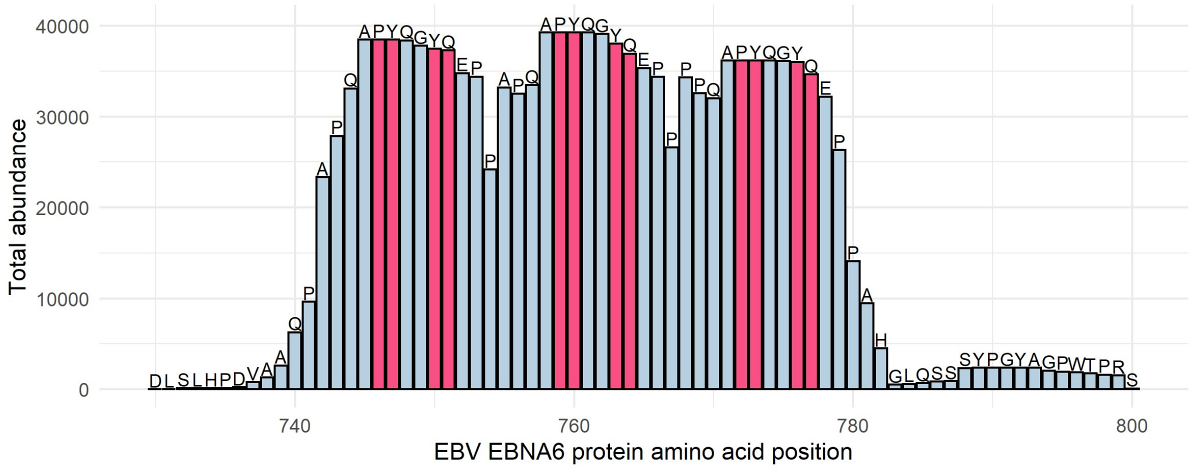


**Figure S9. Epitope B maps to three repeated sequences in EBNA6 protein of EBV, refers to Results chapter under Figure 4.** Altogether 19'718 peptide epitopes containing consensus sequences of clusters comprising epitope B and captured from the current clinical cohort were extracted from the database. Individual peptide abundance values within MVA immunoprofiles were summarized together across the whole cohort (n=96). Peptides were aligned onto EBNA6 protein primary amino acid sequence, with a minimum match of 6 amino acids. To calculate total abundance (*y-axis*), aligned peptide abundances were summarized in the given amino acid positions. Amino acid positions in pink color represent those which are contained in the consensus sequence of epitope B. *y-axis* – total abundance of peptides across clinical cohort aligned onto given amino acid position; *x-axis* –position of 730-800 amino acid fragment of EBV EBNA6 protein; *letters* – one-letter abbreviations of amino acids in given positions; *fill color* – amino acid positions which contain amino acids present in the consensus sequence of epitope B.

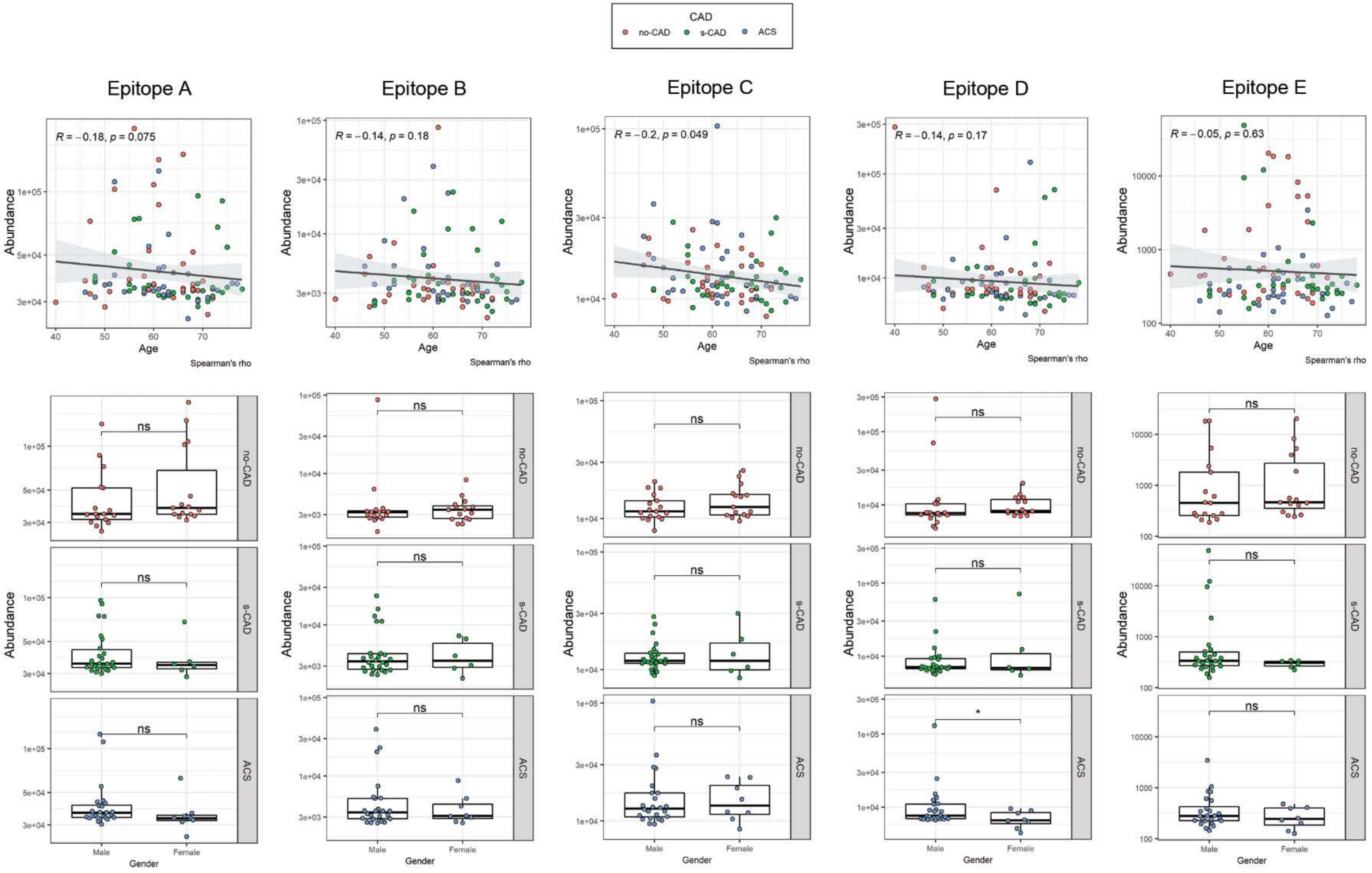


**Figure S10. A weak negative correlation was observed with age and immune response to epitope C, whereas no significant associations were seen with other epitopes with regard to age or gender, refers to Figure 5. Upper scatter plots**: *x-axis* – age, *y-axis* – abundance of peptides containing core epitopes (above scatter plots). R refers to Spearman rho correlation coefficient with p-value

for the coefficient. **Lower boxplots**: pair-wise Mann-Whitney U tests, p-values not adjusted for multiple comparisons, ns $p>0.05$; *x-axis* – male vs female groups; *y-axis* - abundance of peptides containing core epitopes (above scatter plots).

a

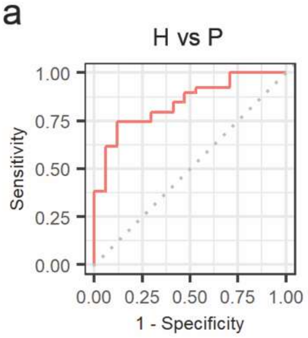


| Parameter | Value |
|---|---|
| AUROC | 0.843 |
| 95% CI | 0.738…0.948 |
| Probability threshold | 0.715 |
| Sensitivity | 74% |
| Specificity | 88% |

b

| Training subset (80%) | | |
|---|---|---|
| *Group* | *Accuracy* | *Balanced accuracy* |
| *H* | 15/17 = 88% | 81% |
| *P* | 29/39 = 74% | |
| **Validation subset (20%)** | | |
| *Group* | *Accuracy* | *Balanced accuracy* |
| *H* | 4/4 = 100% | 84% |
| *P* | 6/9 = 67% | |

**Figure S11. Multivariable model differentiates patients with periodontitis from controls based on immune response to certain epitopes, refers to Figure 5.** A generalized linear model was fit on 80% of subjects (with 5x cross-validation) to classify periodontitis subjects (P, n=39) from controls (H, n=17). **a.** The model's area under the receiver operating characteristic curve (AUROC) was 0.843 with 95% CI (0.738…0.948). Using model's prediction probabilities for subjects in the training set, receiver operating characteristic (ROC) analysis was performed to identify an optimal threshold at 0.715. At that threshold the model's prediction sensitivity within the training set was 74% and specificity 88%. **b.** The balanced accuracy of the model within the training set was 81%. The model was validated on the validation subset (20% of samples, n=4 for H, n=9 for P) with a balanced accuracy of 84%.

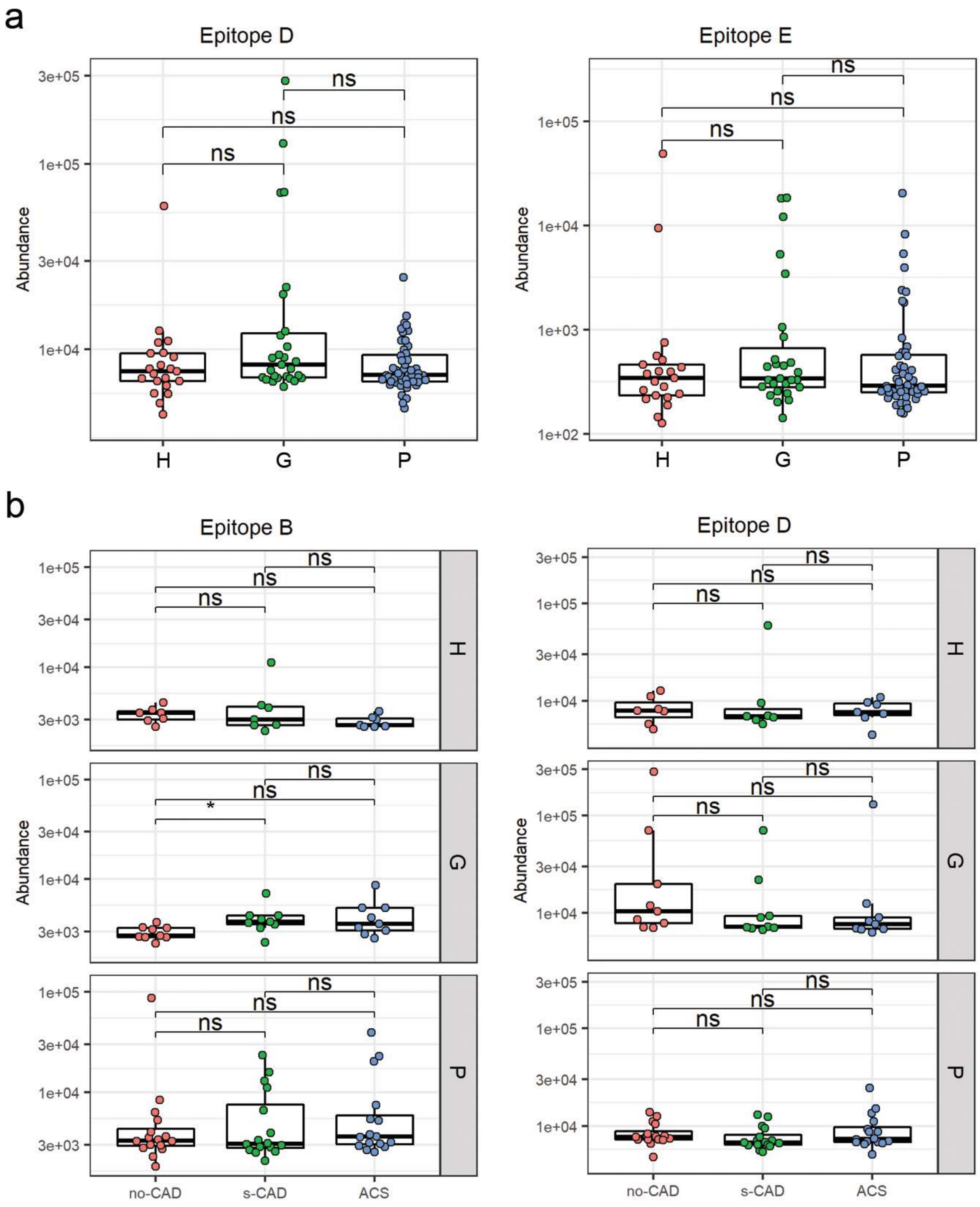


**Figure S12. Response to epitopes D and E was not differentiating between periodontitis groups, similarly response to epitopes B and D was not significantly different between no-CAD and ACS groups, refers to Figure 5. a.** Comparisons of immune response to specific epitopes D and E between periodontitis diagnosis groups: periodontally healthy (H, n=21), gingivitis (G, n=27), or periodontitis (P, n=48). **b.** Comparisons of immune response to epitopes B and D between CAD diagnosis groups: no-CAD (n=32), stable CAD (s-CAD, n=32), or acute coronary syndrome (ACS, n=32). **a-b.** Mann-Whitney U test, p-values not adjusted for multiple comparisons, ns $p>0.05$, * $p<0.05$. *Y-axes* – abundance of MVA peptides containing given epitope core sequences.
