# Supplementary material for: Antibody response to oral biofilm is a biomarker for acute coronary syndrome in periodontal disease
Source: Commun Biol. 2022 Mar 4;5:205. doi: 10.1038/s42003-022-03122-4 (PMC8897497; doi:10.1038/s42003-022-03122-4)
Supplement: Supplementary file 2 — Description of Additional Supplementary Files [file 42003_2022_3122_MOESM2_ESM.pdf]

Description of Additional Supplementary Files

File name: Supplementary Data 1
Description: Alignment load values of epitope alignments to periodontal bacteria proteins, data underlying Figure 2. Forty antigen regions (column FragName, consisting of unique protein identifier and amino acid region) with highest summarised alignment load values are represented per each bacterium species. The type of aligned epitopes (dependent on epitope sequence) is in the Type column, with the possibility of having multiple types or none. Individual samples are designated with "ID" and alignment load values are in the table body in base 2 logarithm.

File name: Supplementary Data 2
Description: Group-wise average abundance values of 62 epitope clusters across different classifiers, data underlying Figure 3a.

File name: Supplementary Data 3
Description: Pearson correlation of 62 epitope clusters based on epitope containing peptide abundance values from immunoprofiles of individual samples, data underlying Figure 3b.

File name: Supplementary Data 4
Description: Alignment values from aligning peptide epitopes to primary sequence of Epstein-Barr virus VP26, data underlying Figure 4a. Numbers 1-176 in top row represent amino acid positions. Cohort (n=96), Ctrl (n=9 EBV-seronegative subjects). Alignment values are in base 2 logarithm.

File name: Supplementary Data 5
Description: Dot ELISA signals in relation to MVA findings, data underlying Figure 4. Individual subjects are in separate rows, dot ELISA signal values are in relative arbitrary units. MVA- - predicted seronegative by MVA for anti-P..T.PR response; MVA+ - predicted seropositive by MVA for anti-P..T.PR response.

File name: Supplementary Data 6
Description: Abundance of peptides containing P..T.PR epitopes from immunoprofiles of individuals of periodontal disease groups, data underlying Figure 4b. Individual subjects are in separate rows.

File name: Supplementary Data 7
Description: Abundance of peptides comprising major epitopes in immunoprofiles of individual patients belonging to different diagnosis groups, data underlying Figure 5. Individual subjects are in separate rows.
