# Supplementary material for: Antibody response to oral biofilm is a biomarker for acute coronary syndrome in periodontal disease
Source: Commun Biol. 2022 Mar 4;5:205. doi: 10.1038/s42003-022-03122-4 (PMC8897497; doi:10.1038/s42003-022-03122-4)
Supplement: Supplementary file 10 — Reporting Summary [file 42003_2022_3122_MOESM10_ESM.pdf]

nature portfolio

Corresponding author(s): Kaia Palm

Last updated by author(s): Dec 17, 2021

Reporting Summary

Nature Portfolio wishes to improve the reproducibility of the work that we publish. This form provides structure for consistency and transparency in reporting. For further information on Nature Portfolio policies, see our Editorial Policies and the Editorial Policy Checklist.

Statistics

For all statistical analyses, confirm that the following items are present in the figure legend, table legend, main text, or Methods section.

n/a	Confirmed	
☐	☒	The exact sample size (n) for each experimental group/condition, given as a discrete number and unit of measurement
☐	☒	A statement on whether measurements were taken from distinct samples or whether the same sample was measured repeatedly
☐	☒	The statistical test(s) used AND whether they are one- or two-sided<br>*Only common tests should be described solely by name; describe more complex techniques in the Methods section.*
☐	☒	A description of all covariates tested
☐	☒	A description of any assumptions or corrections, such as tests of normality and adjustment for multiple comparisons
☐	☒	A full description of the statistical parameters including central tendency (e.g. means) or other basic estimates (e.g. regression coefficient) AND variation (e.g. standard deviation) or associated estimates of uncertainty (e.g. confidence intervals)
☐	☒	For null hypothesis testing, the test statistic (e.g. F, t, r) with confidence intervals, effect sizes, degrees of freedom and P value noted<br>*Give P values as exact values whenever suitable.*
☐	☒	For Bayesian analysis, information on the choice of priors and Markov chain Monte Carlo settings
☐	☒	For hierarchical and complex designs, identification of the appropriate level for tests and full reporting of outcomes
☐	☒	Estimates of effect sizes (e.g. Cohen's d, Pearson's r), indicating how they were calculated

Our web collection on statistics for biologists contains articles on many of the points above.

Software and code

Policy information about availability of computer code

Data collection	No software was used.
Data analysis	Data analysis was conducted with MedCalc® Statistical Software (v.19.7.2) and R Statistical programming language (v.4.0.2) in RStudio environment (v.1.4.1106). Exhaustive sequence pattern search tool SPEXS2 was used (http://egonelbre.github.io/spexs2/) for sequence-based unsupervised clustering of peptide antigens.

For manuscripts utilizing custom algorithms or software that are central to the research but not yet described in published literature, software must be made available to editors and reviewers. We strongly encourage code deposition in a community repository (e.g. GitHub). See the Nature Portfolio guidelines for submitting code & software for further information.

Data

Policy information about availability of data

All manuscripts must include a data availability statement. This statement should provide the following information, where applicable:

- Accession codes, unique identifiers, or web links for publicly available datasets
- A description of any restrictions on data availability
- For clinical datasets or third party data, please ensure that the statement adheres to our policy

The data are not publicly available due to containing information that could compromise research participant privacy/consent. Any materials that can be shared will be released via a material transfer agreement.

Field-specific reporting

Please select the one below that is the best fit for your research. If you are not sure, read the appropriate sections before making your selection.

☒ Life sciences ☐ Behavioural & social sciences ☐ Ecological, evolutionary & environmental sciences

For a reference copy of the document with all sections, see nature.com/documents/nr-reporting-summary-flat.pdf

Life sciences study design

All studies must disclose on these points even when the disclosure is negative.

Sample size	Sample size was determined by the availability of blood samples from the Corogene cohort (references 32-34).
Data exclusions	Based on previously conducted work, signals from peptides deemed as technical background and sequencing errors were excluded from MVA profiles before analysis.
Replication	Reproducibility of Mimotope Variation analysis was confirmed by establishing the correlation coefficient of two replicates as R=0.87 (p<0.0001).
Randomization	Allocation of experimental groups was determined by the diagnosis of the donors.
Blinding	Investigators were not blinded to study samples' origin or nature.

Reporting for specific materials, systems and methods

We require information from authors about some types of materials, experimental systems and methods used in many studies. Here, indicate whether each material, system or method listed is relevant to your study. If you are not sure if a list item applies to your research, read the appropriate section before selecting a response.

Materials & experimental systems

n/a	Involved in the study	
☐	☒	Antibodies
☒	☐	Eukaryotic cell lines
☒	☐	Palaeontology and archaeology
☒	☐	Animals and other organisms
☐	☒	Human research participants
☒	☐	Clinical data
☒	☐	Dual use research of concern

Methods

n/a	Involved in the study	
☒	☐	ChIP-seq
☒	☐	Flow cytometry
☒	☐	MRI-based neuroimaging

Antibodies

Antibodies used	IgG molecules of study participants' blood samples were used as primary antibodies.
Validation	No validation for primary antibodies were performed.

Human research participants

Policy information about studies involving human research participants

Population characteristics	Detailed population characteristics of research participants (including age, gender, BMI, diagnoses) are shown in Table S1.
Recruitment	The recruitment of participants to the COROGENE cohort is detailed in reference 34 and recruitment to the PAROGENE study in reference 32.
Ethics oversight	The study was approved by the Helsinki University Central Hospital ethics committee (approval reference number 106/2007).

Note that full information on the approval of the study protocol must also be provided in the manuscript.
